# Impacts of huanglongbing on fruit yield and quality and on flushing dynamics of Sicilian lemon trees

**DOI:** 10.3389/fpls.2022.1005557

**Published:** 2022-12-05

**Authors:** Juan Camilo Cifuentes-Arenas, Hermes Teixeira de Oliveira, Laudecir Lemos Raiol-Júnior, Everton Vieira de Carvalho, Daniela Kharfan, André Luís Creste, Gerardo Gastaminza, Hernán Salas, Renato Beozzo Bassanezi, Antônio Juliano Ayres, Silvio Aparecido Lopes

**Affiliations:** ^1^ Departamento de Pesquisa e Desenvolvimento, Fundo de Defesa da Citricultura, Araraquara, Brazil; ^2^ Departamento de Fitossanidade, Universidade Estadual Paulista ‘Júlio de Mesquita Filho’, Jaboticabal, Brazil; ^3^ Departamento de Pesquisa e Desenvolvimento. John Bean Technologies Corporation, John Bean Technologies JBT Corporation, Araraquara, Brazil; ^4^ Departamento Agrícola, Agro São José, Rio Claro, Brazil; ^5^ Programa Citrus, Estación Experimental Agroindustrial Obispo Colombres, Las Talitas, Argentina

**Keywords:** *Citrus* sp., HLB, phenology shifts, relative yield, essential oils

## Abstract

**Introduction:**

The severe Asian form of huanglongbing (HLB), a vascular disease associated with the phloem-limited bacterium ‘*Candidatus* Liberibacter asiaticus’, is transmitted by the Asian citrus psyllid (ACP) *Diaphorina citri*. Disease impacts are known for sweet oranges and acid limes but not lemons.

**Methods:**

In a five-year study (2017–2021) we compared yield and fruit quality between naturally-infected and healthy 5-yr-old trees of Sicilian lemon ‘Femminello’, and shoot phenology on both lemon and ‘Valencia’ orange, both grafted onto ‘Swingle’ citrumelo, grown in southeastern São Paulo State, Brazil. HLB severity (percentage of tree canopy area with HLB symptoms) was assessed every 3–4 months, fruit yield and quality in May (2017 to 2019) or June/July (2020-2021), and vegetative and reproductive shoots fortnightly on 50-cm-long branches. The development of ACP on one-year-old seedlings of five lemon varieties, ‘Tahiti’ acid lime, ‘Valencia’ orange, and orange jasmine was evaluated.

**Results:**

Symptoms increased from 11% in 2017 to 64% in 2021, and a monomolecular model estimated 10 years for symptoms to occupy >90% of the tree canopy. On average, production of trees with symptom on 20%, 50% or 80% of the canopy respectively dropped by 18%, 38%, and 53% compared to healthy trees. Fruits of symptomatic branches of lemons were 4.22% lighter and the number of dropped fruits did not correlate with symptom severity. Flushing on symptomatic branches started earlier by 15 to 55 days as compared to the healthy branches of lemon and orange. On diseased trees, vegetative and reproductive shoots respectively increased by 24.5% and 17.5% on lemon and by 67.2% and 70.6% on sweet orange, but fruit set was reduced by 12.9% and 19.7% on lemon and orange trees, respectively. ACP reproduced similarly on all tested plants.

**Discussion:**

The fast symptom progress, significant yield reduction, and earlier flushing on diseased trees, providing conditions highly favorable for the pathogen to spread, reinforce the need of prompt diseased tree removal and frequent ACP preventive control to manage HLB in lemons as in any other citrus crop.

## Introduction

Lemons (*Citrus* ×*limon* (L.) Osbeck) are sour orange-citron hybrids with genetic background of *C. medica* L., *C. reticulata* Blanco. and *C. maxima* (Burm.) Merr. (Wu et al., 2018). They are important sources of essential oils, vitamin C, pectin, dry pulp and other high valued subproducts used in the food, cosmetic and hygiene industries (Guimarães et al., 2010). Their production has been traditionally concentrated in regions with typical Mediterranean basin climate, but also in drier regions of subtropical climate (Barry et al., 2020). Currently, Argentina is the world’s largest producer with an estimated planted area of 66.7 thousand ha, which is equivalent to 50.5% of the total country citrus groves. A total of 1.3 million tons were produced in the 2020/2021 harvest season, with nearly 75% destined to the industry of juice and essential oils (Cocaro, 2021). In Brazil, the planted area with lemons increased by 58.3% in the last four years, approaching to 5.7 thousand ha in 2022 in the main citrus belt of São Paulo and west-southwest Minas Gerais states (Fundecitrus, 2022).

Major threats to Sicilian lemons include the ‘mal secco’ disease (MSD), citrus black spot (CBS) and huanglongbing (HLB). MSD is limited to the Mediterranean basin and is caused by the fungus *Phoma tracheiphila* (Petri) Kantsch., resulting in wilt of branches and dieback of twigs leading to tree death within a few months to years. CBS is spread to most citrus regions except the Mediterranean. It is caused by the fungus *Phyllosticta citricarpa* (McAlpine) Van der Aa, and leads to cosmetic deteriorations and loss of market value of fresh fruits. As a quarantine pest, fruit exportation is severely restricted mainly to the European Union countries (Truter et al., 2007; Migheli et al., 2009; Abbate et al., 2019; EPPO, 2022). HLB is associated with the Gram-negative phloem-limited insect-transmitted bacteria ‘*Candidatus* Liberibacter asiaticus’ (*C*Las), ‘*Ca*. L. americanus’ (*C*Lam) and ‘*Ca*. L. africanus’ (*C*Laf). *C*Las and *C*Lam are transmitted by the Asian citrus psyllid (ACP) *Diaphorina citri* Kuwayama and *C*Laf by *Trioza erytreae* (Del Guercio) (Bové, 2006). *C*Laf is restricted to the Arabian Peninsula and South Africa, *C*Lam to Brazil, and *C*Las, which causes the most severe form of the disease, is widely distributed in Asia and the Americas (EPPO, 2022).

HLB affects all commercial citrus species and hybrids. Damage results from blockage of the phloem vessels, progressive death of roots, yellowing and drop of leaves and fruits (Achor et al., 2010; Folimonova and Achor, 2010; Graham et al., 2013). HLB also causes significant detrimental alterations in various chemical and organoleptic characteristics of the fruit juice (Baldwin et al., 2010; Dala Paula et al., 2018; Dala-Paula et al., 2019). In sweet oranges (*C*. ×*aurantium* L.), fruit production decays sharply as the symptoms invade the canopy, especially on young trees (Bassanezi et al., 2011; Bassanezi, 2018). In Mexican (*C.* ×*aurantiifolia* (Chrism.) Swingle) and Persian (*C.* ×*latifolia* (Yu.Tanaka) Tanaka) limes, yield reduction is apparently less severe than in sweet oranges, with the damages resulting mainly from decreases in number and weight of the fruits present on the symptomatic branches (Flores-Sánchez et al., 2015; Robles-González et al., 2017).

The dynamics of HLB occurrence and progress in the field are strongly associated with the phenology of citrus trees, which is characterized by new shoot flushing cycles (vegetative, reproductive, or mixed) that usually overlap with cycles of new root growth (Bevington and Castle, 1985). These cycles are more well defined in time in the Mediterranean and subtropical regions, and in trees of sweet oranges, than in the tropical climates and in trees of acid limes or lemons (Mendel, 1969). Since new shoots are the preferred sites for feeding, reproduction, and oviposition of ACP (Yamamoto et al., 2001; Pluke et al., 2008; Cifuentes-Arenas et al., 2018), as well as the sites of access of *C*Las into the trees during ACP feeding (Lopes and Cifuentes-Arenas, 2021), flushing cycles influence the population dynamics of the vector and pathogen spread within and between orchards. With respect to new shoots of lemons, responsiveness of adults to volatiles emitted by new shoots of ‘Eureka’ lemon (Patt and Sétamou, 2010), and equivalent reproduction potential of ACP on Sicilian lemons compared to sweet oranges (Alves et al., 2018), have been found. Therefore, during flushing periods the protective measures to reduce the likelihood of infective psyllids reaching the new shoots of healthy plants must be intensified (Bassanezi et al., 2020).

Despite the considerable economic importance of Sicilian lemons, information on damage caused by HLB in this citrus has been scarce compared with other citrus (species and hybrids), especially sweet oranges. No study regarding yield and tree phenology has been carried out. Published studies include reports on symptoms disappearance on trees exposed to high temperatures (Labuschagne and Kotzé, 1988), lack of transmission of *C*Laf through seeds (van Vuuren et al., 2011), detection of *C*Las in the psyllid *Cacopsylla citrisuga* Yang & Li feeding on diseased ‘Eureka’ lemon (Cen et al., 2012), and downregulation of defense-related proteins and increase in zinc concentration in diseased ‘Todo del ano’ lemon, as compared to healthy trees (Nwugo et al., 2013). Contrasting results also have been reported. In short-term greenhouse experiment, the titer of *C*Las in ‘Eureka’ was lower than that in oranges, and leaf symptom had disappeared six months after inoculation (Folimonova et al., 2009), but in long-term field trials the titers and symptom progress in ‘Eureka’ were comparable to those in sweet oranges (Ramadugu et al., 2016). The absence of information on the impacts of HLB on several aspects of Sicilian lemon led us to carry out this work, which was undertaken with the objective to assess the impact of HLB on fruit production and quality of lemons, and on tree phenology of lemon and sweet orange trees.

## Material and methods

### Plants and study site characteristics

This study included adult trees of lemon cultivar ‘Femminello’ and trees of ‘Valencia’ sweet orange, both grafted onto ‘Swingle’ citrumelo (*Citrus ×insitorum* Mabb.), and was carried out in a commercial farm located at the municipality of Rio Claro, southeastern São Paulo state (SPS), Brazil, where estimated 62% of the sweet orange trees in the region were expressing HLB symptoms in 2021 (Fundecitrus, 2021). Both ‘Femminello’ and ‘Valencia’ trees were 5-year-old at the time the evaluations started. The lemon trees were growing in a block of 9.72 ha, located at least 1 km apart from the nearest edge of the farm, where HLB symptoms were initially present on 15% of the trees. The trees had been planted in December 2012 at 2.6 m distance between plants and 7.0 m between rows (ca. 549 plants ha^-1^), which were positioned at 354° of azimuthal orientation. The sweet orange trees of the same age were growing in a 6.1 ha block, planted at 2.5 m between plants and 6 m between rows (ca. 667 plants ha^-1^), with a 6° of azimuthal orientation of the rows and also located at ca. 1 km of the nearest edge of the farm and 0.9 km far from the selected lemon block. All agricultural practices including fertilization, maintenance pruning, phytosanitary sprays (including intensive applications of insecticides every 7 to 15 days for ACP control), and harvest, were carried out by the grower as in the remaining citrus blocks. The climate of the region based on Köppen-Geiger classification is typically Cwa, mesothermic, prevailing hot and humid summers and dry winters. The annual climatological norms are: rainfall of 1.414 mm, average temperature of 21.1°C, wind speed of 1.44 m/s, relative humidity of 68.6% and 2573 hours of sunshine (Alvares et al., 2013). Meteorological conditions were monitored daily using an automatic weather station (iMETOS IMT300-USW, Pessl Instruments, Austria), located ca. 5 km away from the experimental blocks.

### HLB symptom severity and yield loss progress

This study included only ‘Femminello’ lemon trees. An initial survey was carried out to visually estimate the severity of HLB (percentage of HLB leaf symptoms on the tree canopy) of each tree in the block, using as a guide the typical HLB symptoms in four lemon varieties that had been previously characterized (Panccioni, 2015). Tree infection was confirmed through quantitative PCR (qPCR) analysis from eight asymptomatic leaves sampled throughout the canopy of the asymptomatic trees, or eight leaves showing the typical HLB symptoms sampled from symptomatic branches. Sampling processing and qPCR analysis were as described in Murray and Thompson (1980) and Li et al. (2009). For the experiment, 150 trees were selected: 30 qPCR-negative and totally asymptomatic (treated hereafter as “healthy trees”) plus 120 qPCR-positive with symptoms visible from approximate 5 to >90% of the canopy area (treated hereafter as “diseased trees” or “HLB-affected trees”). Every 3 to 4 months, the trees were visited and the area with symptoms was drawn on template figures representatives of the trees (**Supplementary Figure 1**). The figures were then processed using the ImageJ software (Schneider et al., 2012). From the total 120 diseased trees, 32 trees with symptoms present on average 10% of the canopy at the beginning of the study were selected to model symptom progress over time.

**Figure 1:**
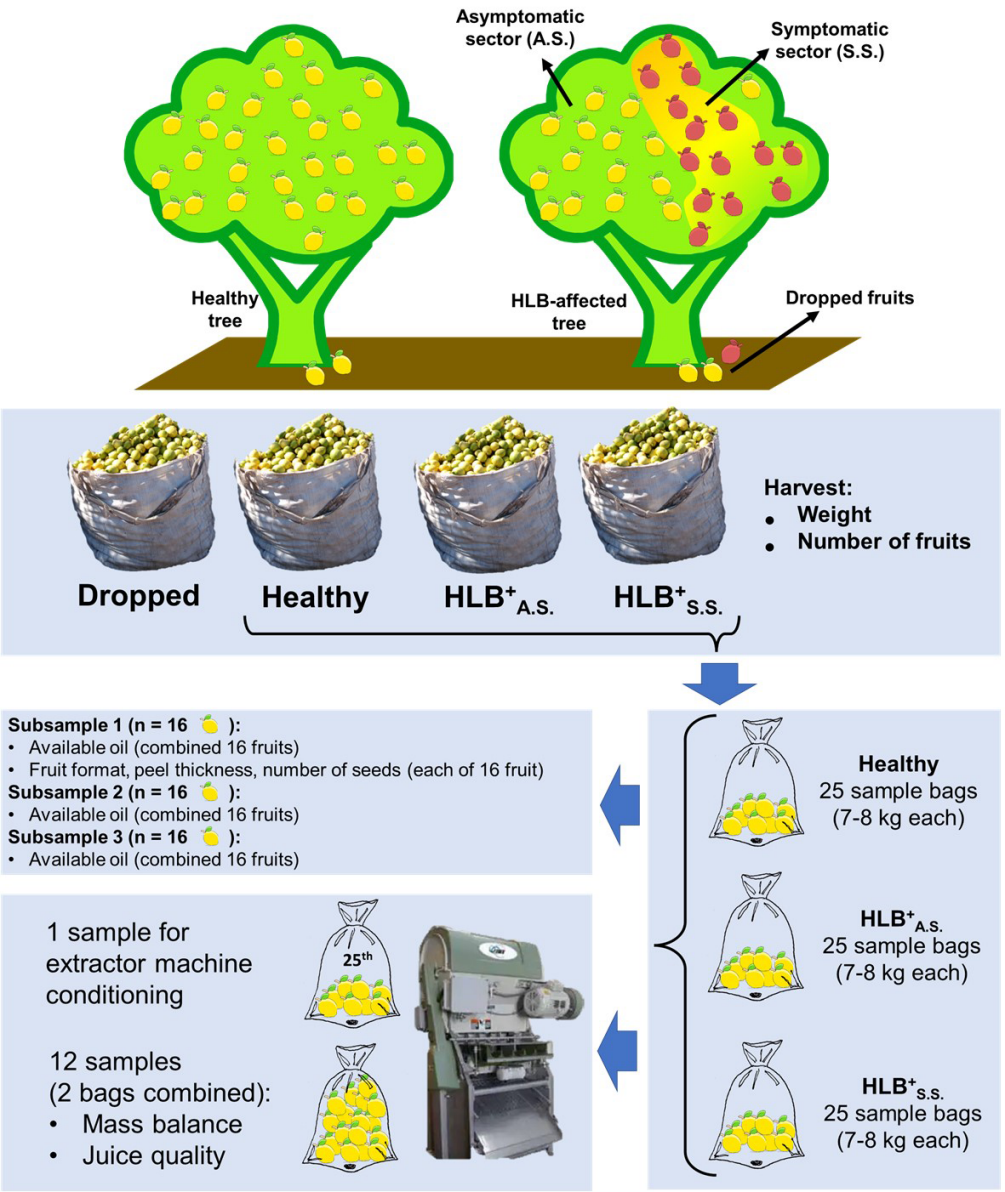
Schematic representation of the workflow from harvest to physical and chemical characterization of fruits harvested from healthy trees and from the asymptomatic (A.S.) and symptomatic (S.S.) sectors of the canopy of HLB-affected trees of the 5-year-old ‘Femminello’ lemon trees used in this work.

For the yield loss progress study, all 150 selected trees were harvested once a year from 2017 to 2021, in the main harvest time of each year. All harvested fruits were placed inside 50 L bags. In some cases, it was necessary to use more than one bag per tree (**Supplementary Figure 2**). On each tree, harvest started by collecting all fruits present on the ground (dropped fruit) that still were suitable for industrial processing. In healthy trees, harvest proceeded by collecting the fruits present on the entire canopy. In HLB-affected trees, fruits from the symptomatic area were collected before those from the asymptomatic area. All the bags of each sector in diseased trees or of the entire canopy in healthy trees were weighed individually to identify the heaviest bag. This heaviest bag was used to determine the number of fruits and to estimate the average weight per fruit. After weight and number evaluations, all fruits were dispensed in a one-ton harvest bag, separated by group (dropped, healthy, asymptomatic, symptomatic; **Figure 1**).

### Physical and chemical characterization of the fruits

Except for the dropped fruits, a total 25 samples of 7 to 8 kg each were taken from each one-ton bags of healthy, asymptomatic and symptomatic fruits, placed inside small bags (sample bags), and sent to laboratory for chemical and physical analyses, as described in **Figure 1**. For chemical analysis, the fruits were processed first from the healthy trees, and then from the asymptomatic and symptomatic branches from the diseased trees. To quantify the amount of available oil, 3 subsamples of 16 fruits each were prepared by taking one fruit per sample bag, randomly distributed from bags 1 to 16 (subsample 1), bags 1 to 7 plus 17 to 25 (subsample 2), and bags 8 to 23 (subsample 3). Before oil quantification, fruit shape (relation between polar and equatorial diameter), peel thickness and number of seeds were determined individually for each fruit of the subsample 1 (**Figure 1**). To determine mass balance and juice quality, the remained fruits were combined in 12 sample bags per treatment, with each bag being a combination of two of the 24 samples. The remained 25^th^ sample of each treatment was used for conditioning and adjustment of the extractor machine (JBT model 391). Mass balance was determined using a balance Toledo model 2090, and consisted of the percentage of the weight of extracted pulpy juice, core, peel, and frit. Fruit juice quality consisted of the amounts of titratable acidity (TA), total soluble solids (TSS), oils, viscosity, the secondary metabolites hesperidin, pectin, and limonin, and TA/TSS ratio.

All juice quality parameters were measured considering the standard methods used by the citrus industry located around the world. TSS was determined by direct measurement using ATAGO refractometer, the TA by titrating the juice with a phenolphthalein and NaOH solutions, the available content of oil in fruit and content of oil in juice by the Scott Method, and the secondary metabolites hesperidin, pectin, and limonin were measured using colorimetric methods (Davis Test) or HPLC. All methods used in this section are described in detail in Redd et al. (1986), Kimball (1991), and JBT (2018).

### Tree phenology

This study included ‘Femminello’ lemon and ‘Valencia’ orange trees. From the total trees used to estimate yield, one subgroup of 10 asymptomatic trees and another of 10 trees with symptoms on >75% of the canopy were selected. On the west and east side of these trees, four branches of 50 cm long located at the approximate center of the respective quadrants were tagged (**Supplementary Figure 1**). On the HLB-affected trees care was taken to select branches expressing HLB symptoms on leaves. Each branch was then monitored every 15 to 21 days to determine presence and number of new shoots and fruits. The vegetative shoots were classified in their stage of development (Cifuentes-Arenas et al., 2018). The reproductive shoots were counted regardless whether they were single-flowered leafless, single-flowered leafy, multiflowered leafless or multiflowered leafy (Agustí et al., 2022). Fruits were counted regardless of their size.

### Reproductive potential of *Diaphorina citri*

This study included one year-old plants of 5 varieties of Sicilian lemon, ‘Valencia’ orange, and orange jasmine (*Murraya paniculata* (L.) Jack), and was conducted in an acclimatized room with the temperature maintained at 26°C ± 2, relative humidity of 65% ± 10, and photoperiod of 12 h of light (300 µmol m^-2^s^-1^), provided by LED lamps, and 12 h of dark. Lemon varieties were ‘Lisbon L8A’, ‘Genova EEAT’, ‘Lisbon Frost’, ‘Eureka Frost’ and ‘Femminello’. Buds of lemons varieties and ‘Valencia’ were grafted on ‘Rangpur’ lime (*C. × otaitensis* (Risso & Poit.) Risso) stems of plants growing in conical tubes of 300 mL containing pine bark as substrate. Orange jasmine seedlings were growing in the same containers. The study was carried out in two stages. In a first stage, the oviposition potential of two unmated 15-day-old ACP couples, confined within a mesh sleeve cage on a new shoot at stage V2 (Cifuentes-Arenas et al., 2018) was evaluated. The presence of eggs was observed every 24 h with the help of a 30× magnifying hand glass. If oviposition was confirmed in one observation, it was assumed that these eggs were laid within the previous 24h, so the adults remained confined for additional 48h, to complete a total of 72h of confinement for mating and oviposition. Then, the adults were removed, the new shoots detached to count the number of laid eggs in the laboratory under a stereoscope. In the second stage, the survival potential of immature ACP was evaluated. For this, the plants were pruned 10-15 days prior to the confinement of up to 30-day-old mated females. Four to five females per new shoot were confined. The new shoots were observed every 2 h until an average number of 40 eggs per shoot (± 10) was identified. Then, the females were removed, in order to guarantee a similar initial egg population and to avoid potential competition for feeding sites of later developed nymphs and adults. Three days later, when the shoot had already grown up to approximately 10 to 20 mm in length, and some of the leaf primordia were open, the number of eggs was counted to confirm the established limit. Seven days after the initial confinement date, the shoots were observed again to count the total number of nymphs (without differentiating instars) and determine egg viability. The plants remained with the new shoots enclosed within a mesh sleeve cage and were observed every 24 h for newly emerged adults, which were removed and counted.

### Data analysis

The data on symptoms severity progress was fitted to the monomolecular growth model *HLB*_*severity*_(*%*)=*A*∗(1−*e* ^(−*k*∗(*t*−*xc*))^) where *A* is the value maximum achievable area of the canopy with symptoms, *xc* a biological constant, and *k* the rate of severity increase as a function of time (*t* in days) (Madden et al., 2007). The data on symptom severity (as the predictor variable) and the relative yield ([yield of symptomatic tree/mean yield of healthy tree]) as the response variable was fitted to the negative exponential model *y*=*e* ^(−β*x)^ where the coefficient *β* is the rate of decay in the relative yield as symptom severity *x* increases (Madden et al., 2007). Linear regression was used to find the increase or decrease rate and determine the relationship between symptom severity and the proportional fruit drop, and between relative number of fruits (fruits from HLB-affected trees/fruits from healthy trees) and relative yield. The data on the influence of HLB on industrial processing parameters and on fruit and juice quality were submitted to analysis of variance (ANOVA) using a generalized linear mixed model (GLMM, *lmer* function in *lme4* package) (Bates et al., 2015), considering the disease status of harvested fruits (healthy, asymptomatic and symptomatic) as a fixed effect and year a random effect. When significant differences were detected, the means were compared by the Tukey-HSD test.

Due to eradication of the ‘Valencia’ block in September 2018, a second block with similar characteristics (age, rootstock variety, planting density, proximity to the farm edges and to the lemon block) was selected in February 2019, resulting in two periods analyzed separately for the phenological study: a first period from the 1^st^ to the 16^th^ assessment date, and a second from the 24^th^ to the 97^th^ date. For each period, data were first transformed into the area under the curve (*AUC*) as previously described (Shaner and Finney, 1977; Madden et al., 2007; Carvalho de et al., 2020; Carvalho et al., 2021), for branch occupancy of vegetative shoots V1/V2 (*AUC_V1V2_*) or reproductive shoots (*AUC_rs_*), and mean number of fruits per branch (*AUC_fruits_*). *AUC* uses the trapezoidal integration method to quantitatively summarize the intensity of a variable of interest over time, like disease progress or flushing dynamics. *AUC* was then submitted to a two-way ANOVA, with citrus species (sweet orange or lemon) and status of HLB (healthy or diseased) as categorical variables. When significant differences were detected, the means were compared by the Tukey-HSD test. Parameters of the biological life cycle of ACP on different rutaceous genotypes were submitted to a one-way ANOVA, with means compared by the Tukey-HSD test. Significant level of *p* = 0.05 and R software (R Core Team, 2020) were used for all analysis.

## Results

### Weather conditions

The averages of air temperature were 21.5°C, 22.9°C, 22.4°C, 21.2°C and 20.8°C in 2017, 2018, 2019, 2020 and 2021, respectively. The warmest months were November and March in 2017 and 2018 (avgs. 24.5 and 26.2°C), and January in 2019 to 2021 (avgs. 26.5, 24.2, 24.3°C, respectively). July was the coldest month in 2017 (avg. 16.8°C) and 2019 to 2021 (avgs. 17.2, 17.5, 14.7°C), and August in 2018 (avg. 19.1°C). The average yearly cumulative rainfall during the 5 years of study was 1251.1 mm (**Table 1**). In 2017 and 2020 the cumulative rainfalls were by 9.6% and 27% higher and in 2018, 2019 and 2021 were by 21.5%, 1.9%, and 13.1% lower than the average of the five years of study, respectively (**Table 1**). During the period of low temperatures and rain (April/May to July/August), symptoms of water deficiency (wilting of leaves, loss of brightness of the leaf surface and, in extreme cases, shedding of leaves and dieback of the terminal twigs) were detected first on healthy trees of both lemon and orange.

**Table 1:** Summary of weather conditions recorded from 2017 to 2021 in southeastern São Paulo State, in the area of study.

Climate variables	2017	2018	2019	2020	2021	Averages
Temperature (°C)						
Average	21.5	22.9	22.4	21.2	20.8	21.8
Maximum^(a)^	36.6^(Jan)^	34.9^(Mar)^	33.8^(Oct)^	34.0^(Sep)^	34.1^(Sep)^	34.7
Minimum^(a)^	10.7^(Jul)^	11.1^(Jul)^	9.5^(Jul)^	8.8^(May)^	5.2^(Jul)^	9.1
Relative humidity (%)						
Average	67.2	70.6	74.4	74.8	77.9	73.0
Maximum^(a)^	96.3^(May)^	92.7^(Jan)^	95.2^(Mar)^	100.0^(Dec)^	100.0^(Jan)^	96.8
Minimum^(a)^	23.0^(Oct)^	29.9^(Jul)^	39.6^(Oct)^	25.1^(Sep)^	24.5^(Sep)^	28.4
Rainfall (mm)						
Year	1371	982	1228	1589	1087	1251.1
Maximum^(a)^	374^(Jan)^	233^(Oct)^	250^(Feb)^	385^(Feb)^	251^(Oct)^	298.7
Minimum^(a)^	11.1^(Jun)^	0.0^(Apr)^	0.0^(May)^	7.5^(Apr)^	6.6^(Aug)^	5.0
Feb. to May	501	195	469	609	363	427.2

a Superscript in parenthesis correspond to the month when the average of daily max. or min. temperatures or the cumulative rain of the month was recorded.

**Figure 2:**
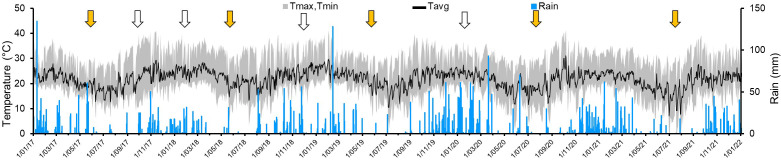
Daily records of temperatures (average: black line; amplitude: gray area) and rainfall (black bars) recorded in the farm located in the municipality of Rio Claro, SPS, Brazil, used to assess yield and flushing on 5-year-old ‘Femminello’ lemon and ‘Valencia’ sweet orange trees. Weeks of main and secondary fruit harvests are indicated by yellow and white arrows, respectively.

For lemon trees growing under SPS subtropical conditions the critical months for fruit development are February to May. It is worth noting that this time-period of 2018 was particularly dry, when rainfall was by 54.4% lower than the average 427.2 mm for the years 2017 to 2021 (**Figure 2**; **Table 1**). Yearly average of relative air humidity varied from 67% to 78%, with the lowest daily average records occurring mostly between July and October (**Table 1**). **Supplementary Table 1** shows more detailed meteorological data.

### Progress of HLB severity

**Figure 3:**
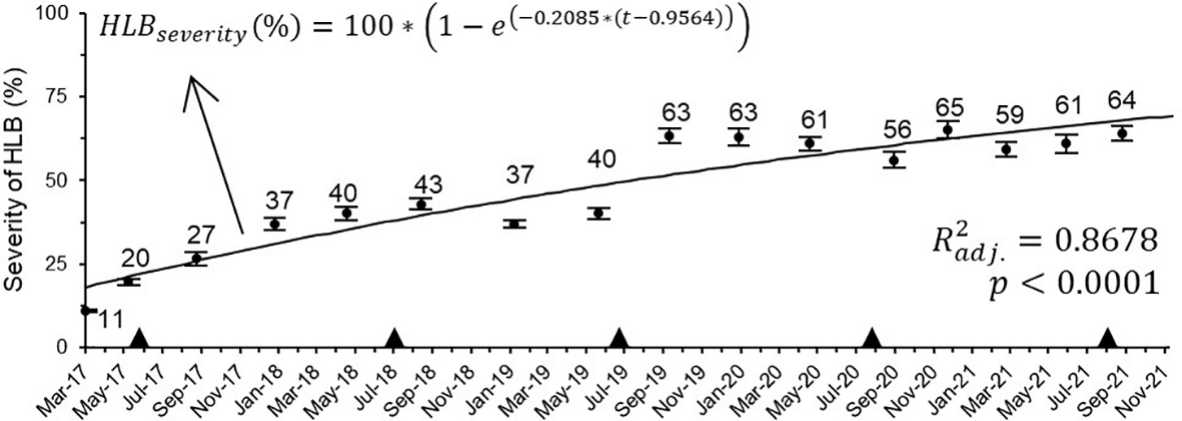
Progress of HLB severity on 5-year-old ‘Femminello’ lemon trees (n = 34; 1^st^ assessment = 24/03/17). The continuous black line is the estimated progress based on the given model equation. The black triangles indicate the time when the trees were pruned (top or lateral), normally within 30 days after the main harvest date of each season.

Evaluations began in the 3rd week of April 2017, 4 weeks before the first main harvest. At this time, symptoms were observed on average 11.1% ± 0.32 of tree canopy area. Disease severity progressively increased until September 2018 (1.5 year later), when reached a maximum of 42.9% ± 1.7. In the following evaluations, symptom decreased by 6.2% in January 2019, and subsequently increased by 3.7% until May 2019. From October 2019 to December 2021, the last evaluation date, symptomatic area ranged from minimum 56% to maximum 65% (**Figure 3**).

The data on symptoms progress significantly fitted the monomolecular model (*xc* = -0.9564 ± 0.2843; *k* = 0.2084 ± 0.0215) (**Figure 3**). As predicted by the model, under the conditions where this study was carried out, it would take on average 2.4 (2.3 to 2.7), 5.7 (5.2 to 6.8), and 10.1 (8.8 to 12.3) years for HLB symptoms to spread to approximately 10 to 50, 75, and 90% of an adult lemon tree canopy, respectively, which, at the beginning of this study, was ca. 4.4 m high and 4.1 m wide, resulting in a canopy volume of 46.9 m^3^.

### Influence of HLB on yield

Five main harvest seasons were evaluated: the first three at the end of May 2017, 2018, and 2019, the fourth in the last week of July 2020, and the last in the third week of July 2021. Harvest of 2020 was delayed due to disruptions in the production chain caused by Covid-19 pandemics, and of 2021 to unfavorable weather conditions causing severe flower and fruit drop. Yield in healthy trees averaged 82.3 kg per tree (min.: 34.6 ± 3.93 kg in 2018; max.: 105.11 in 2020), and in diseased trees, regardless symptom severity, averaged 57.78 kg (min.: 25.13 ± 1.23 kg in 2018; max.: 68.5 ± 2.43 kg in 2017).

**Figure 4:**
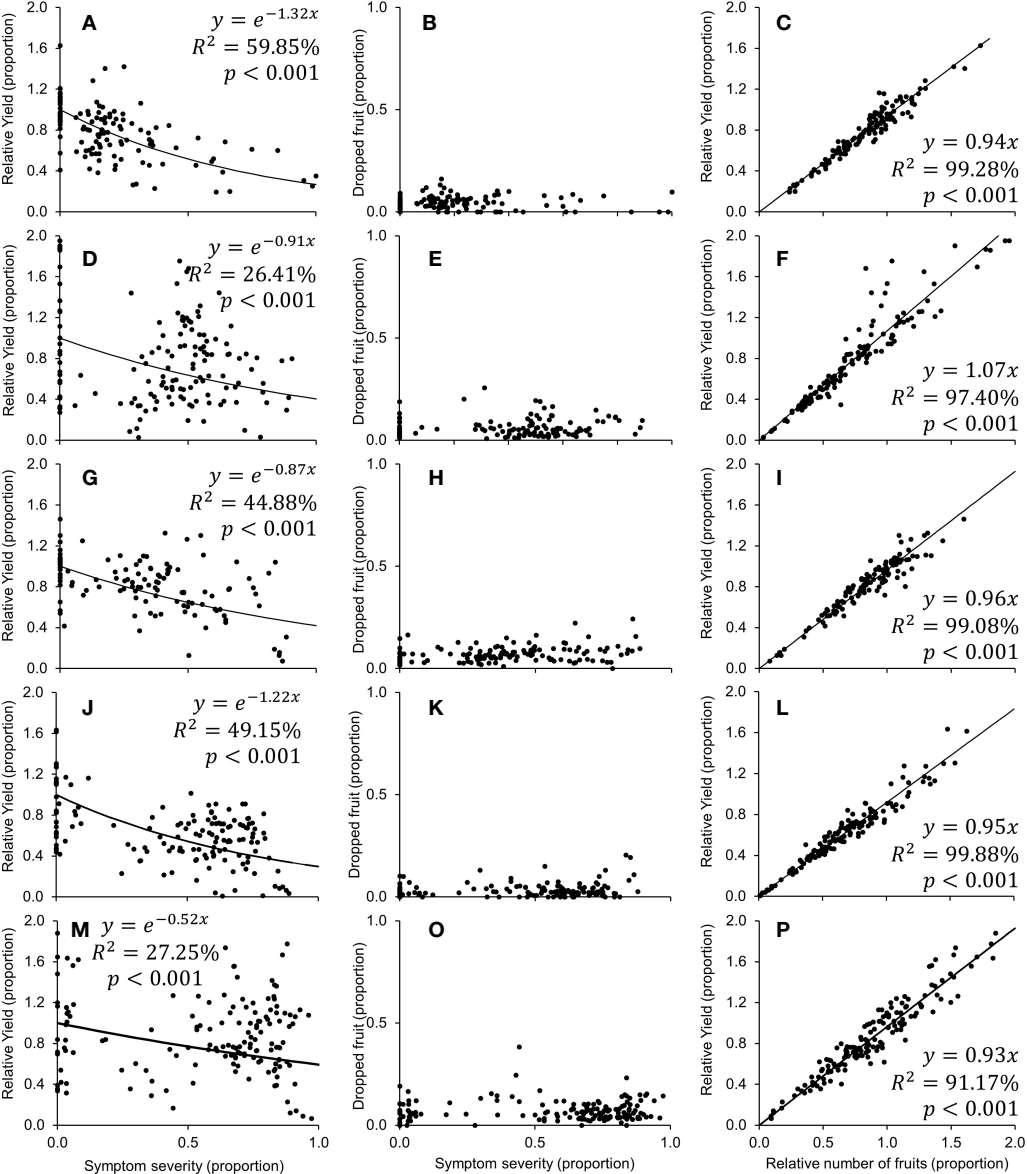
Relationship between symptom severity and relative yield [(fruit from HLB^+^ tree)/(fruit from healthy tree)] **(A, D, G, J, M)** or dropped fruit **(B, E, H, K, O)**, and between the relative number of fruits [(number of fruits from HLB^+^ trees)/(number of fruits from healthy trees)] and relative yield **(C, F, I, L, P)**, calculated from the 2017 **(A–C)**, 2018 **(D–F)**, 2019 **(G–I)**, 2020 **(J–L)**, and 2021 **(M–P)** harvest season of 5 year-old ‘Femminello’ lemon trees.

The negative exponential model adequately described the relationship between relative yield ([kg fruit from symptomatic trees]/[kg fruit from healthy trees]) and symptom severity for each harvest season (**Figures 4A, D, G, J, M**). The rate of decay (*β*) in fruit production averaged -0.99 (min.: -1.32 in 2017; max.: -0.52 in 2021; **Figure 4**). For diseased trees with 20, 50, 80 and 100% of leaf symptom on the canopy, the model estimated average relative yields of 76.7 to 90.1%, 51.6 to 77.2%, 34.7 to 65.9% and 26.6 to 39.6%, respectively (**Table 2**). Preharvest fruit drop averaged 5.84% (min.: 4.12% ± 0.34 in 2017; max.: 7.62% ± 0.41 in 2021; **Figure 4**), and did not correlate with symptom severity in any harvest season. The relative yield correlated positively with the relative number of fruits ([number of fruits in diseased trees]/[number of fruits in healthy trees]) and showed an average increase rate of 0.97 (min.: 0.93 in 2021; max.: 1.07 in 2018; **Figure 4**).

**Table 2:** Estimated averages and range of relative yield^a^ of ‘Femminello’ lemon trees exhibiting variable severity levels of HLB leaf symptoms on the canopies, as projected by the negative exponential model shown in **Figure 3**.

Harvest season	Symptom severity			
	20%	50%	80%	100%
2017	76.74 (67.51–87.23)	51.59 (37.45–71.06)	34.68 (20.77–57.89)	26.61 (14.02–50.49)
2018	83.41 (79.37–87.66)	63.54 (56.13–73.24)	48.41 (39.69–60.76)	40.38 (31.50–53.64)
2019	84.01 (81.13–87.00)	64.70 (59.28–70.61)	49.82 (43.32–57.30)	41.86 (35.14–49.85)
2020	78.39 (75.29–81.61)	54.40 (49.18–60.17)	37.75 (32.13–44.36)	29.59 (24.19–36.20)
2021	90.08 (87.67–92.56)	77.02 (71.97–82.43)	65.85 (59.08–73.40)	59.32 (51.80–67.94)
Averages	82.53 (78.19–87.21)	62.25 (54.80–71.50)	47.30 (39.00–58.74)	39.55 (31.33–51.63)

Values of relative yield^a^ (proportion 0 to 1) are expressed in percentage (0% to 100%).

### Influence of HLB on industrial processing parameters and on fruit and juice quality

The different components of fruit mass balance was influenced by the incidence of HLB (**Table 3**). Fruits collected from symptomatic branches of HLB-affected trees had significantly less percentage of peel (-4.31%), core (-1.89%) and frit (-5.54%), but more pulpy juice (+3.04%) than fruits collected from healthy trees (**Table 3**).

**Table 3:** Mass balance (percentages) of ‘Femminello’ lemon fruits from healthy trees and from the asymptomatic and symptomatic sector of HLB-affected trees (diseased trees) collected in the southeastern São Paulo State (average values of five harvest seasons – 2017 to 2021) ^yz^.

	n	Peel (%)	n	Core (%)	n	Frit (%)	n	Pulpy juice (%)
**Disease status**								
Healthy	59	18.57 ± 0.37a	59	15.84 ± 0.35a	59	10.10 ± 0.29a	59	55.49 ± 0.28b
Asymptomatic	60	18.32 ± 0.32ab	60	15.98 ± 0.37a	60	9.84 ± 0.20ab	60	55.82 ± 0.29b
Symptomatic	60	17.77 ± 0.37b	60	15.54 ± 0.29b	60	9.54 ± 0.26b	60	57.17 ± 0.20a
**Anova results**								
DF		2, 173		2, 173		2, 173		2, 173
*F* value		4.2766		6.3280		6.7293		20.0430
*p*(>*F*)		0.0154		0.0022		0.0015		<0.0001

y Values with the same lowercase (within column) letters, did not differ significantly (p > 0.05).

z Detailed average values by harvest season are shown in **Supplemental Table 2**.

For physical characterization, the relationship between polar and equatorial diameters (fruit shape), peel thickness, number of seeds, amount of available oil and weight were determined (**Table 4**). HLB did not affect peel thickness (avg. 5.1 ± 0.07 mm) or fruit shape. Fruits from symptomatic branches contained by 18% less seeds and by 1.77% less available oils in the peel than fruits from healthy trees. Infection by *C*Las also reduced significantly fruit weight. Fruits from HLB-affected trees were, on average, by 4.22% lighter than those from healthy trees (**Table 4**).

**Table 4:** Physical characterization of ‘Femminello’ lemon fruits from healthy trees and from the asymptomatic and symptomatic sector of HLB-affected trees (diseased trees) collected in different harvesting seasons in southeastern São Paulo State ^yz^.

		n	Fruit shape	n	Peel thickness (mm)	n	Number of seeds	n	Available oil (%)	n	Fruit weight (g)
**Disease status**										
Healthy	84	1.17 ± 0.01a	84	5.19 ± 0.12a	84	7.04 ± 0.35a	13	6.79 ± 0.13a	149	117.2 ± 4.42a
Asymptomatic	84	1.18 ± 0.01a	84	5.10 ± 0.11a	84	7.01 ± 0.30a	13	6.72 ± 0.20ab	452	112.5 ± 5.01b
Symptomatic	84	1.17 ± 0.01a	83	5.01 ± 0.14a	84	5.76 ± 0.33b	13	6.67 ± 0.21b	506	112.4 ± 5.53b
**ANOVA results**										
DF	2, 245		2, 245		2, 245		2, 32		2, 1100	
*F* value	0.6062		0.4590		5.3408		3.5193		6.3412	
*p*(>*F*)	0.5462		0.6325		0.0054		0.0416		0.0018	

y Values with the same lowercase (within column) letters, did not differ significantly (p > 0.05).

z Detailed average values by harvest season are shown in **Supplemental Table 3**.

The extracted juice also was analyzed for the main quality parameters and for the content of three secondary metabolites (**Tables 5**, **6**). Levels of juice TA, content of TSS, TA/TSS ratio and viscosity were, respectively, by 3.05%, 6.18%, 3.58%, and 6.71% significantly lower in fruits collected from symptomatic sectors of HLB-affected trees canopies than from healthy trees (**Table 5**). The oil content in extracted juice was by 20.43% higher in symptomatic fruits than asymptomatic or healthy fruits (**Table 5**). The infection by *C*Las did not impact the content of pectin. However, the levels of hesperidin in fruits harvested from asymptomatic and symptomatic branches, and of limonin in fruits from symptomatic branches were significantly increased by 8.69%, 18.59% and 37.64%, respectively, compared to fruits from healthy trees (**Table 6**).

**Table 5:** Industrial quality of extracted ‘Femminello’ lemon juice from fruits collected from healthy trees and from the asymptomatic and symptomatic sector of HLB-affected trees (diseased trees) collected in different harvesting seasons in the south-southeast of São Paulo State ^xyz^.

		n	TA (%)	n	TSS	n	Ratio	n	Oils (%)	n	Viscocity (cP)
**Disease status**										
Healthy	60	6.23 ± 0.06a	60	10.52 ± 0.19a	60	1.68 ± 0.02a	21	0.07 ± 0.01b	51	6.26 ± 0.22a
Asymptomatic	60	6.20 ± 0.08a	58	10.16 ± 0.20b	60	1.64 ± 0.02b	21	0.07 ± 0.01b	50	6.28 ± 0.29a
Symptomatic	60	6.04 ± 0.07b	58	9.87 ± 0.25c	59	1.62 ± 0.02b	20	0.08 ± 0.01a	51	5.84 ± 0.20b
**ANOVA results**										
DF	2, 173		2, 169		2, 172		2, 56		2, 145	
*F* value	8.2397		29.8190		10.3110		10.1610		4.3969	
*p*(>*F*)	0.0004		<0,0001		<0.0001		0.0002		0.0140	

x TA: titrable acidity; TSS (total soluble solids content).

y Values with the same lowercase (within column) letters, did not differ significantly (p > 0.05).

z Detailed average values by harvest season are shown in **Supplemental Table 4**.

**Table 6:** Content of secondary metabolites of extracted ‘Femminello’ lemon juice from fruits collected from healthy trees and from the asymptomatic and symptomatic sector of HLB-affected trees (diseased trees) collected in different harvesting seasons in the south-southeast of São Paulo State ^yz^.

		n	Hesperidin	n	Pectin	n	Limonin
**Disease status**						
Healthy		52	313.38 ± 19.25 c	22	314.40 ± 16.08 a	22	19.79 ± 1.38 b
Asymptomatic		52	340.61 ± 20.58 b	22	313.72 ± 19.04 a	22	24.84 ± 1.86 ab
Symptomatic		52	371.64 ± 19.38 a	22	317.76 ± 21.30 a	22	27.24 ± 2.19 a
**ANOVA results**						
DF			2, 149		2, 59		2, 59
*F* value			15.267		0.0351		9.1771
*p*(>*F*)			<0.0001		0.9655		0.0003

y Values with the same lowercase (within column) letters, did not differ significantly (p > 0.05).

z Detailed average values by harvest season are shown in **Supplemental Table 4**.

### Influence of HLB on tree phenology

**Figure 5:**
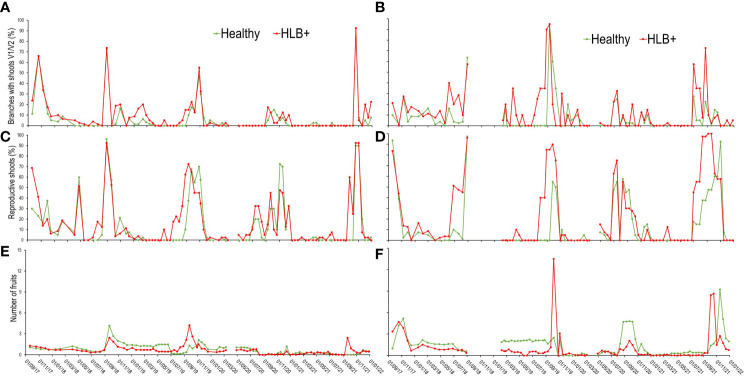
Patterns of the occupancy dynamics of new shoots in stages V1/V2 **(A, B)**, reproductive shoots **(C, D)**, and of the number of fruits **(E, F)** per tree. Each data point corresponds to the averages of 10 plants from the 1^st^ (19/1/2017) to the 23^rd^ (21/1/19) evaluation date, and of 5 plants from the 24^th^ (7/2/19) to the 97^th^ (27/12/19), in ‘Femminello’ lemon **(A, C, E)** and ‘Valencia’ sweet orange **(B, D, F)** trees.

For healthy le mon trees, five main vegetative flushing peaks, mostly with new flush growths at V1 and V2 stages of development, were observed on the tree canopies (**Figure 5A**), namely, in October 2017, end of August 2018, between August and September 2019, September and December 2020, and October and December 2021. In SPS these time-periods generally coincide with the transition from late winter to early or end of spring. HLB-affected trees (red lines) also flushed in the same periods, but in October 2018, August/September 2019, and September 2021 peaks started one to three weeks earlier. The flushing that begun in early December 2018 extended until February 2019 (**Figure 5A**), and those of September 2019 and 2020 extended until December of the same years, both on healthy and HLB-affected trees. In the case of the main flushing of 2019, HLB-affected trees began flushing almost a month earlier than the healthy trees.

Flushing was more frequent on ‘Valencia’ orange (**Figure 5B**) than on lemon, but the main peaks coincided in both citrus types (**Figure 5A**), generally concentrating at the end of winter and during all spring. The healthy trees did not flush between March and May 2019, when new shoots were found on average 14% of the branches of the diseased trees. As on lemon, flushing started earlier on diseased ‘Valencia’ trees, and was more evident from late autumn to mid-winter of 2019, when flushing started 5 assessment dates (55 days) earlier than on healthy trees (**Figure 5B**).

In the case of reproductive shoots, the differences observed between diseased and healthy trees were similar for both citrus types (**Figures 5C, D**). On lemon, the emission of reproductive shoots occurred two and five assessment dates (30 to 55 days) earlier on HLB-affected than on healthy trees, which generally coincided with mid to late winter or early spring of each year (**Figure 5C**). On ‘Valencia’, despite the less frequent initiation of shoot emission as compared to lemon, a greater intensity of reproductive shoots was found on plants with HLB (**Figure 5D**).

The number of fruits per branch was also counted on lemon trees. From October 2017 to April 2018 fruit number gradually increased on healthy trees (**Figure 5E**), but not on diseased trees despite two minor blooms (**Figure 5C**), an indication that fruits from secondary blooms rarely set on diseased trees. Fruit set from the second largest bloom identified between April and July 2018 was low (**Figure 5E**), both on diseased and healthy trees. Although in August-September 2018 blooming intensity on healthy trees was similar to that on diseased (**Figure 5E**), fruit set was by 22.5% lower on diseased trees. In the main bloom of 2019 (September), the maximum number of fruits recorded per branch was by 47.9% lower on healthy than on HLB-affected trees. However, after the natural physiological fruit drop, the number of fruits per branch was lower on diseased than on healthy trees. On diseased trees the final fruit number was reduced by only 11.7%. In contrast, in 2021, the bloom that began in July and lasted until November (**Figure 5E**) generated a lower number of fruits on diseased trees. In the case of ‘Valencia’ sweet orange trees, the number of fruits per branch was always lower on diseased than on healthy trees, with the exception for October 2019 and 2021 (**Figure 5F**), when the number of fruits on diseased trees was respectively ca. 5 and 3.5 times higher than on healthy trees. These two periods corresponded to two large blooms (**Figure 5D**), but fruit set observed in the following assessments was lower on diseased than on healthy trees.

**Figure 6:**
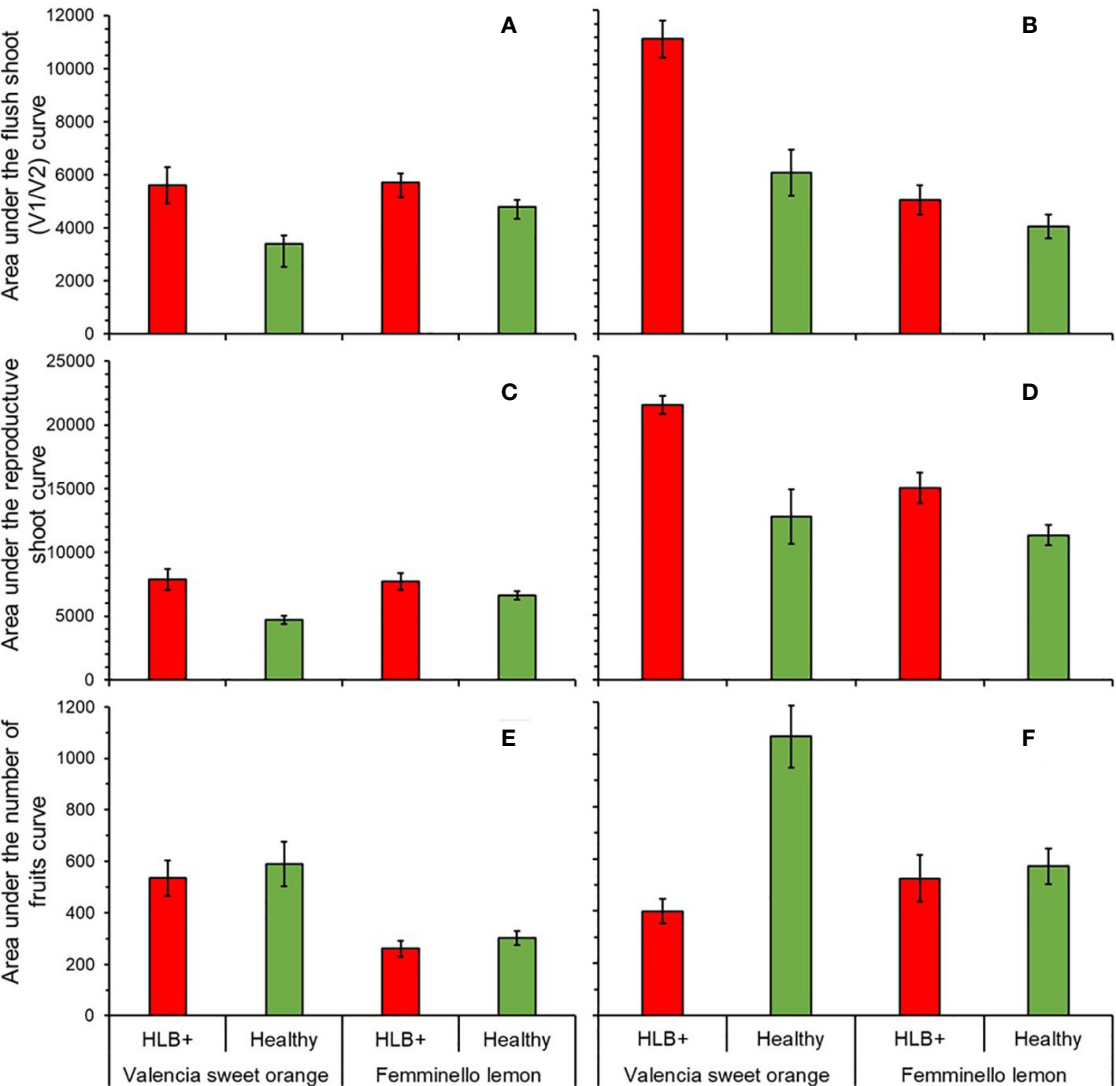
Area under the curve of the percentage of branches with vegetative new shoots in stages V1 and V2 **(A, B)**, reproductive shoots **(C, D)** and number of fruits per branch **(E, F)**, from the 1^st^ to 16^th^ **(A, C, E**; n = 10) and 24^th^ to 97^th^ (**B, D, F**; n = 5) assessment dates in ‘Femminello’ lemon and ‘Valencia’ sweet orange trees.

In the first period (1^st^ to 16^th^ assessment), the *AUC_V1V2_* differed between citrus types (F_1, 36_ = 5.12, p = 0.0435) and between disease status (F_1, 36_ = 12.56, p = 0.0011). Interaction (citrus type x disease status) was not significant (F_1, 36_ = 3.14, p = 0.0851). *AUC_V1V2_* was by 12.7% higher in orange than in lemon trees, and by 35.6% higher in HLB-affected than in healthy trees (**Figure 6A**). In the second period (**Figure 6B**), the interaction between citrus type and disease status was significant (F_1, 16_ = 9.09, p = 0.0082). In HLB-affected trees, *AUC_V1V2_* of sweet orange was by 111.79% higher than in lemons (F_1, 16_ = 39.31, p < 0.0001), while no difference was detected in healthy trees between species (F_1, 16_ = 4.02, p = 0.0622) (**Figure 6B**). In ‘Valencia’, *AUC_V1V2_* was by 78.8% higher in HLB-affected than healthy trees (F_1, 16_ = 27.08, p = 0.0001), while in ‘Femminello’ *AUC_V1V2_* in healthy trees did not differ from that in HLB-affected trees (F_1, 16_ = 0.88, p = 0.3615) (**Figure 6B**).

The dynamics of branch occupancy by reproductive shoots (either single/multi-flowered leafless or leafy shoots) was also studied (**Figures 6C, D**). In the first period, interaction between plant type and disease status was significant (F_1, 36_ = 7.00, p = 0.0120), so each factor was analyzed within the other. In HLB-affected trees *AUC_rs_* of ‘Valencia’ did not differ from that on ‘Femminello’ (F_1, 36_ = 0.36, p = 0.5539), while in healthy trees, ‘Femminello’ *AUC_rs_* was by 57.7% higher than that of ‘Valencia’ (F_1, 36_ = 9.88, p = 0.0033) (**Figure 6C**). In the second period (from the 24^th^ to the 97^th^ assessment), *AUC_rs_* of ‘Valencia’ was 26.0% higher than in ‘Femminello’ (F_1, 16_ = 6.98, p = 0.0177), and 48.0% higher in HLB-affected than healthy trees (F_1, 16_ = 19.76, p = 0.0004) (**Figure 6D**).

In the first period, the mean number of fruits per 50-cm-long branch was by 119.2% higher in ‘Valencia’ than in ‘Femminello’ (F_1, 36_ = 31.91, p < 0.0001), but did not differ between diseased and healthy trees (F_1, 16_ = 1.73, p = 0.1970) (**Figure 6E**). No significant interaction (citrus type x disease status) was observed (F_1, 16_ = 0.42, p = 0.5210). In the second period (**Figure 6F**), interaction between citrus type and disease status was significant (F_1, 16_ = 23.20, p = 0.0002). In ‘Valencia’, the *AUC_fruits_* was by 66.9% lower in diseased than in healthy trees (F_1, 16_ = 9.65, p = 0.0068), while in ‘Femminello’ the *AUC_fruits_* did not differ between healthy and diseased trees (F_1, 16_ = 0.01, p = 0.9270). In healthy trees, *AUC_fruits_* was by 126.2% higher in ‘Valencia’ than in ‘Femminello’ (F_1, 16_ = 31.39, p < 0.0001). In diseased trees, no difference was observed between ‘Valencia’ and ‘Femminello’ (F_1, 16_ = 1.46, p = 0.2444) (**Figure 6F**).

### Potential of different lemon varieties for *Diaphorina citri* reproduction

In general, no significant differences were found for egg number (F_7,72_ = 1.043, p = 0.409), and eggs (F_7,68_ = 0.628, p = 0.731), nymphs (F_7,68_ = 0.639, p = 0.722) and total viability (F_7.68_ = 0.494, p = 0.836) for different lemon varieties, ‘Tahiti’ lime, ‘Valencia’ orange and *M. paniculata* (**Table 7**). On average, 42 eggs had been laid by the females at the end of the 72h-confinement period. Of these eggs, an average of 84.9% hatched to first-instar nymphs, and 84.2% of the nymphs reached the adult stage. Overall, 71.8% of nymphs that hatch from eggs became adults.

**Table 7:** Parameters of the life cycle of *Diaphorina citri* on different lemon varieties, ‘Tahiti’ lime, ‘Valencia’ orange and *Murraya paniculata*.

Genotype	n	Number of eggs	n	Egg viability (%)	n	Nymph viability (%)	n	Egg-to-adult viability (%)
Eureka Frost	10	44.5 ± 4.4 A	9	88.3 ± 2.2 A	9	81.2 ± 4.4 A	9	72.3 ± 5.3 A
Lisbon Frost	10	39.7 ± 2.9 A	10	83.0 ± 1.8 A	10	83.6 ± 4.3 A	10	69.8 ± 4.5 A
Genova EEAT	10	43.4 ± 3.0 A	10	82.5 ± 3.5 A	10	84.3 ± 3.7 A	10	69.8 ± 4.3 A
Lisbon LSA	11	43.8 ± 3.1 A	9	85.8 ± 1.8 A	9	80.3 ± 6.9 A	9	68.6 ± 6.3 A
Femminello	10	35.4 ± 3.8 A	9	83.7 ± 2.4 A	9	84.6 ± 6.0 A	9	71.1 ± 5.6 A
‘Tahiti’ lime	10	41.5 ± 5.2 A	10	84.6 ± 2.0 A	10	82.0 ± 3.4 A	10	69.7 ± 3.6 A
‘Valencia’ orange	10	40.0 ± 2.8 A	10	85.3 ± 2.2 A	10	91.8 ± 1.7 A	10	78.4 ± 3.5 A
*M. paniculata*	9	47.7 ± 2.6 A	9	86.4 ± 2.0 A	9	86.2 ± 5.2 A	9	74.5 ± 4.4 A

Values with the same letter did not differ significantly (p > 0.05), Tukey-HSD test.

## Discussion

The objective of this work was to study the progress and impact of HLB symptom severity on the production and quality of fruits and on shoot phenology of adult trees of Sicilian ‘Femminello’ lemon and ‘Valencia’ sweet orange in Brazil, over a period of five years. Disease severity occurred in a cyclical-incremental way; generally increasing from the end of autumn to the end of winter, and slightly declining during the spring and summer (**Figure 3**). This may be related to climate variation impacting flushing and symptom expression. Usually, the lower temperatures and rainfalls during the autumn and winter (**Table 1**, **Figure 2**) seem to inhibit new vegetative flushing and favor symptom expression. Subsequently, after the first rains and increase in temperature that occur at the end of winter to early spring, new flushes emerge, but remain asymptomatic until they become mature and express the typical mottling, which results mainly from the accumulation of starch on the parenchymatic leaf cells (Achor et al., 2010). This seasonal fluctuation on overall symptom expression also was observed in ‘Valencia’ (Montesino, 2011), and seems to be related to the cycles of emission of new flushes which are initially asymptomatic, but as the new tissues mature the typical HLB symptoms appear. This fluctuation can make the work of finding and eliminating diseased trees by scouting teams more difficult depending on the time of the year that scouting is made. Under SPS conditions, April/May to August/September (mid-autumn to late winter/early spring) is the best time-period to detect new symptomatic plants in orange orchards (Belasque Júnior et al., 2009).

Another reason for the variation in HLB severity progress on the lemon trees used in this study could be the practice of pruning of branches located on top and lateral parts of the trees, which is carried out yearly mainly to reduce tree size and regulate fruit production (García, 2001). In the block where this work was undertaken, the trees were pruned within the first 2 weeks from the main harvest period (black triangles in **Figure 3**). The removal of the terminal branches may then have reduced the symptomatic area. At the same time, this practice may have stimulated pathogen migration and symptom expression in the formerly asymptomatic parts of the canopy. In sweet oranges, the HLB pathogen seems to migrate preferentially towards new tissues, which was experimentally demonstrated for *C*Las in top- and root-pruned potted plants in greenhouse (Raiol-Júnior et al., 2021b), and observed for *C*Lam in branch- and trunk-pruned adult field trees (Lopes et al., 2007).

The monomolecular model adequately described the progression of severity over time (p < 0.0001, R^2^ = 86.8%) (**Figure 3**). The rate of increase of symptoms on the canopy of lemon trees (*β* = 0.21) was 31.25% faster than the average reported for sweet oranges (Bassanezi, 2018). The increase in symptoms on tree canopy may have resulted most probably from the internal movement of *C*Las within the tree, starting from the symptomatic areas registered at the beginning of the experiment (Raiol-Júnior et al., 2021b; Raiol-Júnior et al., 2021a).

Similar to other citrus types, HLB significantly impacted fruit yield in lemon trees. The rate of decay in the relative fruit yield as a function of the leaf symptom severity varied between years (**Figures 4 A, D, G, J, M**). The average rate of -0.97 ± 0.14 was similar to the observed averages for the African citrus greening on ‘Valencia’ orange in Swaziland (Catling and Atkinson, 1974), and for the Asian HLB in Mexican lime in Mexico (Robles-González et al., 2017), but lower than the average -1.85 ± 0.06 for the combined data for early-, mid- and late-season sweet orange varieties affected by the Asian HLB in SPS (Bassanezi et al., 2011). Even in 2017, when the decay was steepest (*β* = -1.32 ± 0.33; **Figure 4A**), the rate was 32% lower than the slowest decay reported for the late-season ‘Valencia’ (*β* = -1.66 ± 0.09) (Bassanezi et al., 2011). This shows a less severe impact of HLB on yield loss in lemons than in sweet oranges. However, the faster symptom progress in lemon than in sweet orange trees may result in equivalent overall negative impacts of the disease on both hosts.

The impact of HLB on lemon fruits was significant but lower than in oranges. Average weight per fruit from asymptomatic or symptomatic branches of HLB-affected trees was, on average, by 4.22% lower than the average weight of fruits from healthy trees. This impact is much lower than the 18.3% to 30.8% reduction observed in sweet oranges (Bassanezi et al., 2009; Bassanezi et al., 2011), or the 9.2% to 18% reduction in ‘Mexican’ and ‘Persian’ limes (Flores-Sánchez et al., 2015; Robles-González et al., 2017). On lemons, no correlation was detected between fruit drop and symptom severity (**Figures 4B, E, H, K, O**), an indication that the main cause for the dropped fruit could be a decrease in fruit set and increase of the post-blossom physiological fruitlet drop, a natural phenomenon that occurs after a profuse flowering to adjust crop load and avoid carbohydrate depletion (**Figures 5C, E**).

Although not investigated, the lower impacts of HLB on lemon fruits in relation to other citrus types could be related, at least in part, to changes in anatomical and physiological traits. It is known that among the most important alterations caused by HLB on citrus trees is the plugging of the phloem vessels by callose deposition, with a subsequent increase in starch content impairing fruit filling (Folimonova et al., 2009; Achor et al., 2010; Folimonova and Achor, 2010). Recently, Sivager et al. (2021) compared the more HLB-tolerant ‘Persian’ with the less tolerant ‘Mexican’ lime and found that the tolerance was associated to a better performance of transpiration, photosynthesis and stomatal conductance, and to a larger pore and phloem cell size, which would result in less accumulation of starch and less vessels clogging.

Similar to some productivity parameters, most physical and chemical quality parameters in lemon fruits were barely affected by HLB or were affected at levels much lower than in sweet oranges (Bassanezi et al., 2009). There was a reduction in TA (-3.05%) and TSS (-6.18%), but an increase in limonin (+37.64%), and hesperidin (+18.59%). The reductions in TA and TSS were similar to those in ‘Persian’ limes (Flores-Sánchez et al., 2015), and the increase in limonin and hesperidin much lower than the 2.6- and 7-fold observed in sweet oranges (Chin et al., 2014; Dala Paula et al., 2018). Pectin content varied between harvest seasons, in a way similar to the observed for sweet orange, but was not impacted by HLB (Baldwin et al., 2010). The reduction in TA and the increase in hesperidin and limonin can represent a deterioration in the quality of the juice, giving it a more bitter taste (Dala Paula et al., 2018).

HLB caused important alterations in the flushing dynamics and fruit occurrence of the tagged branches (**Figure 5**). When both diseased and healthy plants were simultaneously flushing, the number of vegetative or reproductive shoots was higher on diseased trees in ca. of 68% of the assessments (**Figures 5A–D**). Increase in number of new shoots in HLB-affected lemon or sweet orange trees has not been reported so far. However, it has been found that in the initial stages of *C*Las infection, when the pathogen has not even been detected in the roots, the emission of new roots is stimulated (Pulici et al., 2022). The stimulus to flushing could be due to a disruption of transport and accumulation of carbohydrate caused by *C*Las infection (Folimonova et al., 2009; Achor et al., 2010; Cimò et al., 2013). Buildup of carbohydrate could create conditions similar to the practice of girdling, widely used to increase flowering, fruit set and size, in citrus and other plant species (Erner, 1988; Williams and Ayars, 2005; Rivas et al., 2007; López et al., 2015). However, the increase in floral shoots caused by HLB in both lemon and sweet orange did not translate into a greater number of fruits. Instead, there was a reduction in fruit set and an increase in post blossom physiological fruitlet drop (**Figure 6**). This is not surprising considering that the roots are the main sources of carbohydrates to sustain flowering and fruiting (Dovis et al., 2014), and that, in HLB-affected trees, root deterioration is progressive and rapid, starting even before the symptoms appear on the upper scion (Graham et al., 2013; Johnson et al., 2014).

In addition to increase in flushing, in some periods of the year flushing started earlier on HLB-affected than healthy trees (by ca. 15 to 55 days) in both lemon and sweet orange. The earlier flushing was more likely to occur between mid/end autumn to end winter/early spring (**Figure 5**), when temperatures and rainfall were lower (**Table 1**, **Figure 2**), which could have important ecological implications. The timing of the onset of flushing events represents an important evolutionary feature, common to many deciduous tree species. Most of the available studies on pathogen-induced phenology shifts in woody plants report severe premature shedding of leaves in deciduous trees as a protective mechanism of the plant to prevent the spread of non-vascular pathogens (Burdon and Bartlett, 2022). However, in the case of citrus infected by *C*Las, an evergreen plant species and a vascular pathogen, the shift in flushing does not seem to result from a direct and exclusive action of the pathogen, as described, but rather from an indirect response of the tree to changes in its relationship with the soil water availability that, ultimately, seems to favor pathogen dispersion. The level of water deficit that stimulates leaf shedding and flushing shifts in diseased citrus trees should be studied in future experiments, since it could allow estimating at what periods of the year diseased trees growing in areas outside the farms are more likely to flush. This would help to control ACP with the use of insecticides or massive release of natural vector enemies.

Evidence of changes in water relationship of citrus plants by *C*Las infection have been already reported. In HLB-affected trees leaf area and shoot size are reduced and evapotranspiration and sap flow are, on average, 29% and 58% lower than in healthy trees, respectively (Carvalho de, 2022; Keeley et al., 2022). This indicates that diseased trees use water more efficiently and have a lower requirement for the available water in the soil than healthy trees (Hamido et al., 2017). Under SPS climatic conditions, it is common to see healthy plants in non-irrigated commercial groves beginning to show symptoms of water deficiency from April/May onwards. Such symptoms include wilting of leaves, loss of leaf surface brightness and, in extreme cases, shedding of leaves and dieback of the terminal twigs. These leaf symptoms are not commonly observed on HLB affected trees. The leaves fall, but much later in the dry season, while those on healthy trees remain withered, still attached to the branches. Leaf drop on the diseased tree stimulates the emission of new shoots. All these field observations were confirmed in the present work, and has serious implications for the HLB epidemics, since it creates favorable conditions for psyllid to lay eggs and reproduce, first on the diseased trees (Cifuentes-Arenas et al., 2018). In the absence of ACP control measures, and considering the high reproductive potential of the psyllids in the genotypes tested (**Table 6**), the offspring of these psyllids can survive within the grove for several weeks during the late autumn to spring (Ashihara, 2004) until suitable climatic conditions allow healthy plants to flush and favor pathogen spread, thus increasing the risk of new infections (Lopes et al., 2017; Lopes and Cifuentes-Arenas, 2021). This reinforces the importance of eliminating symptomatic trees both within the grove and in the neighboring areas, in addition to frequent vector control, to reduce the chances of *C*Las dissemination (Bassanezi et al., 2013; Bassanezi et al., 2020).

## Conclusion

Although the impacts of HLB on physical and chemical characteristics of ‘Femminello’ lemon fruits were lower than recorded for sweet orange varieties in several other publications, the overall damage caused by the disease was remarkable. Also, earlier flushing on diseased trees may provide conditions highly favorable for the pathogen to spread. All this should break down the empirically constituted grower’s idea that HLB in lemon is a disease of minor importance, and highlight the importance to take the measures to control HLB in lemons as rigorously as in any other citrus crop.

## Data availability statement

The original contributions presented in the study are included in the article/**Supplementary Material**. Further inquiries can be directed to the corresponding author.

## Author contributions

SL and JC-A designed the study. GG, HS, and AA managed the resources for the financing of the project. JC-A, HO, LR-J, EC, and DK collected the data. JC-A performed the statistical analysis. JC-A and SL contributed to interpretation and drafting the article. JC-A, SL, LR-J, EC, RB, GG, HS, AA, and AC contributed to critical revision of the manuscript. All authors contributed to the article and approved the submitted version.

## Funding

The present work was financed in part by Fundecitrus, Brazil (project FDC #041630), and by a joint partnership between Fundecitrus, Brazil, and the Estación Experimental Agroindustrial Obispo Colombres (EEAOC) and Asociacion Tucumana del Citrus (ATC), Argentina.

## Acknowledgments

We thank the JBT Corporation for supporting the fruit quality analysis and the Agro São José group for allowing us to carry out the research on one of their groves. We also thank George Andrew Charles Beattie, from Western Sydney University, for the critical review and valuable comments on this manuscript.

## Conflict of interest

Author AC was employed by the company Agro São Jose. Author DK was employed by the company JBT Corporation.

The remaining authors declare that the research was conducted in the absence of any commercial or financial relationships that could be construed as a potential conflict of interest.

## Publisher’s note

All claims expressed in this article are solely those of the authors and do not necessarily represent those of their affiliated organizations, or those of the publisher, the editors and the reviewers. Any product that may be evaluated in this article, or claim that may be made by its manufacturer, is not guaranteed or endorsed by the publisher.
