# Supplementary material for: Impacts of huanglongbing on fruit yield and quality and on flushing dynamics of Sicilian lemon trees
Source: Front Plant Sci. 2022 Dec 5;13:1005557. doi: 10.3389/fpls.2022.1005557 (PMC9760907; doi:10.3389/fpls.2022.1005557)
Supplement: Supplementary file 1 [file DataSheet_1.docx]

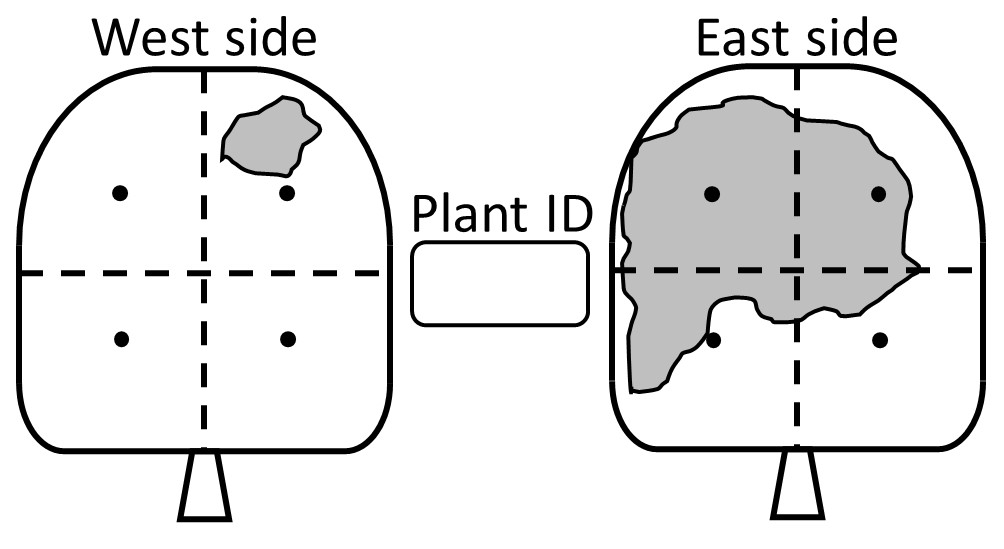


Supplementary Figure 1. Template figure used to represent the canopies of the trees (outer continuous lines) and to draw the proportional area expressing symptoms of HLB (gray areas). Crossed dashed lines represent visual division of the sectors (quadrants) of the canopies, and the black dots represent the approximate location where 50-cm-long branches were tagged for evaluation of flushing dynamics.


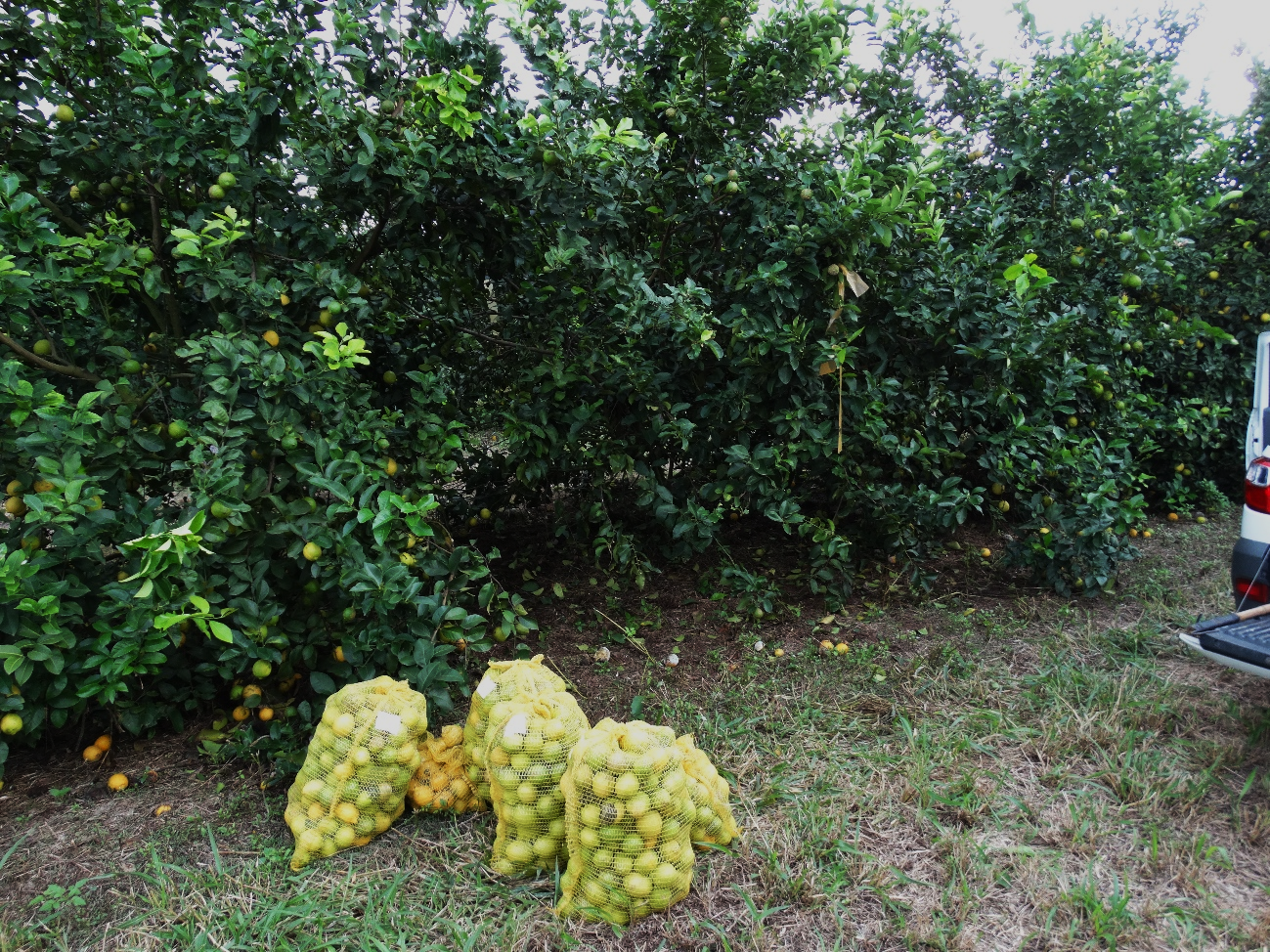


**C**

**A**

**Tree harvested**

**iii**

**i**

**ii**


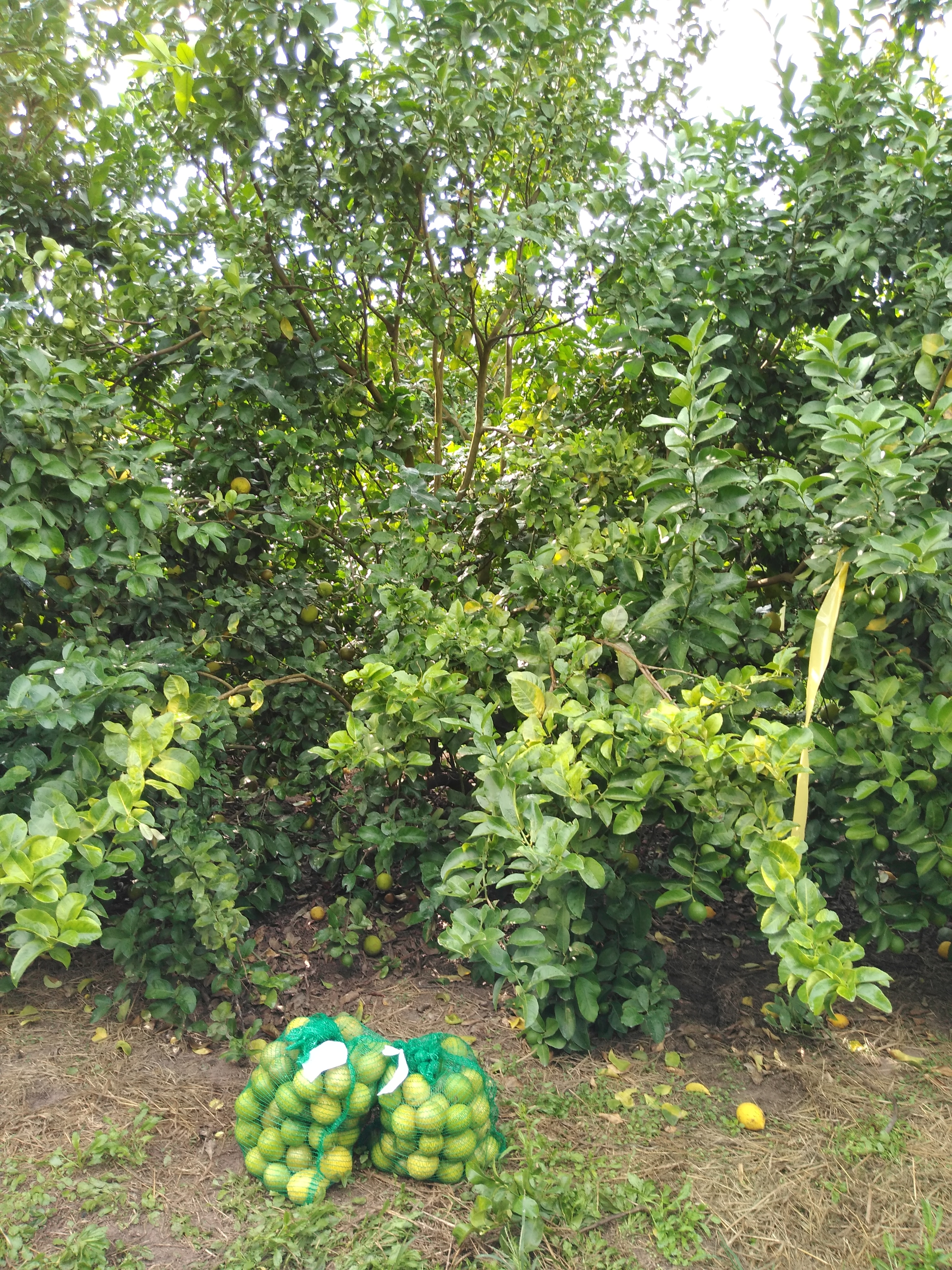

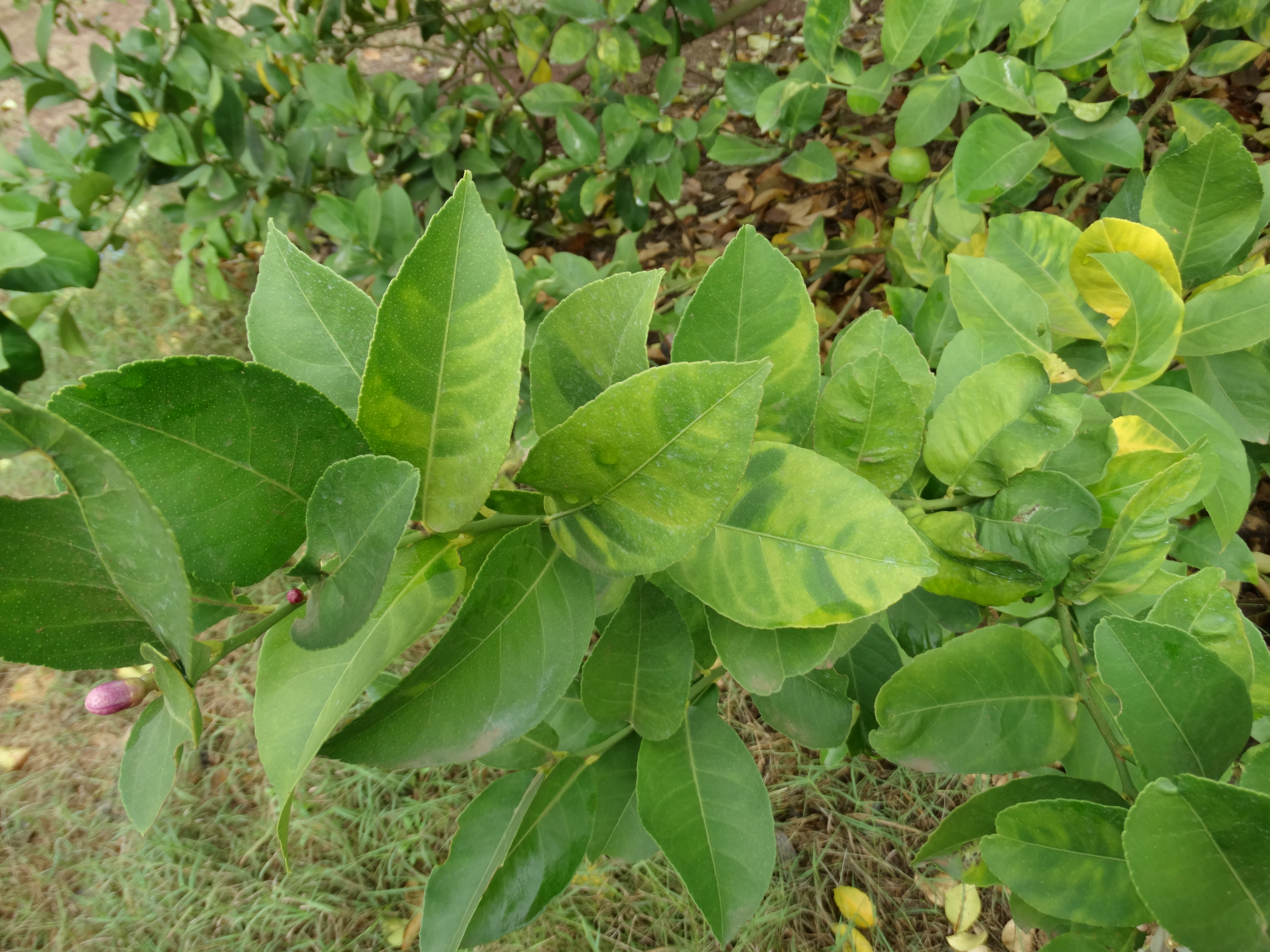


**B**

Supplementary Figure 2. Tagged ‘Femminello’ lemon trees after being harvested showing the bags used to collect dropped fruits (i), fruits from the symptomatic branches (ii) and fruits from the asymptomatic branches (iii) (A) and detail of symptomatic leaves (B and C).
