# Supplementary material for: Impacts of huanglongbing on fruit yield and quality and on flushing dynamics of Sicilian lemon trees
Source: Front Plant Sci. 2022 Dec 5;13:1005557. doi: 10.3389/fpls.2022.1005557 (PMC9760907; doi:10.3389/fpls.2022.1005557)
Supplement: Supplementary file 2 [file DataSheet_2.docx]

| Supplementary Table 1.  Monthly averages of the daily records of temperature, relative air humidity [avg. (max-min)] and cumulative rainfall during the course of the experiment | | | | | | | | | | | | | | | | | |
| --- | --- | --- | --- | --- | --- | --- | --- | --- | --- | --- | --- | --- | --- | --- | --- | --- | --- |
| Month | Temperature (°C) | | | | |  | Relative air humidity (%) | | | | |  | Rainfall (mm) | | | | |
|  | 2017 | 2018 | 2019 | 2020 | 2021 |  | 2017 | 2018 | 2019 | 2020 | 2021 |  | 2017 | 2018 | 2019 | 2020 | 2021 |
| January | 23.1 (29.8-19.2) | 25.2 (33.6-19.7) | 26.5 (33.5-21.7) | 24.2 (32.0-19.0) | 24.3 (32.4-19.4) |  | 54.2 (79.8-24.7) | 73.1 (89.7-49.4) | 71.4 (86.5-50.0) | 80.8 (93.8-59.4) | 85.4 (100-48.3) | | 374 | 175 | 87 | 312 | 152 |
| February | 24.3 (33.0-18.6) | 24.4 (32.5-19.5) | 24.5 (31.2-19.8) | 23.1 (29.7-19.2) | 23.2 (31.4-17.5) |  | 66.4 (86.5-38.7) | 78.4 (92.7-55.5) | 78.9 (92.4-58.7) | 86.4 (95.4-69.4) | 84.6 (100-48.1) | | 71 | 85 | 250 | 385 | 202 |
| March | 22.9 (31.0-17.9) | 26.2 (34.9-20.3) | 23.9 (30.8-19.3) | 22.9 (31.7-16.4) | 23.3 (31.8-17.6) |  | 73.3 (86.6-52.2) | 74.1 (92.2-49.2) | 81.3 (95.2-58.0) | 76.8 (94.2-52.0) | 84.8 (100-46.8) | | 159 | 63 | 68 | 169 | 128 |
| April | 21.1 (28.4-16.4) | 23.6 (33.0-16.0) | 23.1 (30.4-17.9) | 20.0 (29.6-12.7) | 20.0 (28.7-12.9) |  | 71.3 (86.6-50.1) | 68.2 (90.0-42.4) | 79.9 (94.9-55.6) | 74.5 (93.4-48.7) | 80.7 (100-42.6) | | 124 | 0 | 150 | 8 | 9 |
| May | 19.5 (26.7-15.0) | 20.7 (30.7-12.7) | 21.1 (28.6-15.6) | 16.5 (26.5-8.8) | 18.0 (27.8-10.5) |  | 83.1 (96.3-61.2) | 68.1 (90.5-40.1) | 79.4 (93.4-55.4) | 72.3 (91.7-44.0) | 80.4 (100-39.8) | | 148 | 46 | 0 | 47 | 24 |
| June | 17.9 (26.5-12.7) | 20.4 (29.8-13.9) | 19.4 (27.1-13.1) | 18.9 (27.5-12.9) | 16.8 (25.9-10.0) |  | 76.8 (93.3-50.7) | 70.4 (89.2-43.4) | 72.7 (89.9-48.0) | 77.6 (92.8-53.1) | 83.5 (100-45.0) | | 11 | 3 | 13 | 109 | 44 |
| July | 16.8 (26.2-10.7) | 20.3 (32.2-11.1) | 17.2 (26.9-9.5) | 17.5 (27.8-9.5) | 14.7 (26.2-5.2) |  | 68.3 (88.0-41.7) | 58.6 (84.8-29.9) | 69.9 (91.5-40.2) | 69.8 (90.2-41.2) | 68.1 (98.4-25.1) | | 27 | 3 | 23 | 14 | 19 |
| August | 19.4 (27.2-13.6) | 19.1 (28.9-12.3) | 18.6 (28.5-10.5) | 18.0 (28.2-9.9) | 19.4 (29.4-10.8) |  | 66.1 (85.2-44.5) | 70.1 (88.4-45.4) | 68.3 (90.6-40.7) | 70.1 (97.9-32.3) | 64.1 (93.4-30.2) | | 37 | 116 | 4 | 46 | 7 |
| September | 21.1 (31.2-13.7) | 22.1 (31.6-14.7) | 22.1 (32.2-14.4) | 23.0 (34.0-13.9) | 23.3 (34.1-14.5) |  | 59.3 (81.4-35.1) | 67.1 (87.3-42.8) | 68.3 (88.3-43.1) | 61.4 (96.1-25.1) | 62.2 (97.0-24.5) | | 37 | 68 | 65 | 9 | 10 |
| October | 24.3 (36.6-14.7) | 23.6 (31.8-18.0) | 24.3 (33.8-16.4) | 23.8 (32.7-16.6) | 21.3 (29.5-15.7) |  | 49.6 (77.7-23.0) | 74.4 (89.9-52.8) | 66.4 (90.0-39.6) | 67.3 (95.2-36.1) | 83.8 (100-50.1) | | 140 | 233 | 158 | 43 | 251 |
| November | 24.5 (33.6-17.9) | 23.9 (31.1-18.5) | 23.8 (31.9-17.7) | 22.5 (31.9-14.8) | 22.6 (30.8-15.5) |  | 66.1 (87.7-41.2) | 74.7 (89.4-55.0) | 77.4 (92.9-56.1) | 73.2 (99.9-35.1) | 75.6 (100-42.2) | | 150 | 177 | 185 | 182 | 122 |
| December | 23.5 (32.4-17.3) | 25.4 (34.3-19.1) | 24.4 (31.6-18.7) | 23.4 (31.2-18.6) | 22.9 (30.8-16.8) |  | 72.3 (90.2-48.2) | 70.2 (89.6-44.8) | 79.0 (93.7-59.2) | 86.9 (100-51.5) | 81.0 (100-48.5) | | 95 | 13 | 225 | 264 | 118 |
| Year | 21.5 (30.2-15.6) | 22.9 (32-16.3) | 22.4 (30.6-16.2) | 21.2 (30.2-14.4) | 20.8 (29.9-13.9) |  | 67.2 (86.6-42.6) | 70.6 (89.5-45.9) | 74.4 (91.6-50.4) | 74.8 (95.1-45.7) | 77.9 (99.1-40.9) |  | 1371 | 982 | 1228 | 1589 | 1087 |
|  | | | | | | | | | | | | | | | | | |

| Supplementary Table 2. Mass balance (percentages) of ‘Femminello’ lemon fruits from healthy trees and from the asymptomatic and symptomatic sector of HLB-affected trees (diseased trees) collected in different harvest seasons in the southeastern São Paulo State. | | | | | | | | | | | |
| --- | --- | --- | --- | --- | --- | --- | --- | --- | --- | --- | --- |
| **Parameter** | **n** | **2017** | **n** | **2018** | **n** | **2019** | **n** | **2020** | **n** | **2021** | **Average** |
| Peel (%) |  |  |  |  |  |  |  |  |  |  |  |
| Healthy | 12 | 18,2±0,3 | 11 | 22,7±0,5 | 12 | 16,0±0,2d | 12 | 15,9±0,3d | 12 | 20,0±0,4 | **18,6±0,4** |
| Asymptomatic | 12 | 17,5±0,2 | 12 | 22,2±0,5 | 12 | 15,7±0,3d | 12 | 15,7±0,4d | 12 | 20,4±0,4 | **18,3±0,4** |
| Symptomatic | 12 | 18,0±0,1 | 12 | 20,8±0,8 | 12 | 16,1±0,2 | 12 | 15,2±0,4d | 12 | 18,6±0,3 | **17,8±0,3** |
| **Average** | **36** | **17,9±0,1** | **35** | **21,9±0,4** | **36** | **16,0±0,1** | **36** | **15,6±0,2** | **36** | **19,7±0,2** |  |
| Core (%) |  |  |  |  |  |  |  |  |  |  |  |
| Healthy | 12 | 14,2±0,3 | 11 | 11,7±0,2d | 12 | 18,5±0,2 | 12 | 18,6±0,1 | 12 | 16,2±0,2 | **15,8±0,4** |
| Asymptomatic | 12 | 14,5±0,2 | 12 | 13,0±0,2d | 12 | 18,7±0,2 | 12 | 18,4±0,1 | 12 | 15,3±0,1 | **16,0±0,3** |
| Symptomatic | 12 | 13,8±0,2 | 12 | 11,3±0,2d | 12 | 18,2±0,2 | 12 | 18,7±0,2 | 12 | 15,7±0,2 | **15,5±0,4** |
| **Average** | **36** | **14,2±0,1** | **35** | **12,0±0,2** | **36** | **18,5±0,1** | **36** | **18,6±0,1** | **36** | **15,8±0,1** |  |
| Frit (%) |  |  |  |  |  |  |  |  |  |  |  |
| Healthy | 12 | 11,9±0,3 | 11 | 8,2±0,3 | 12 | 12,1±0,3 | 12 | 10,7±0,3 | 12 | 7,6±0,1 | **10,1±0,3** |
| Asymptomatic | 12 | 11,5±0,1 | 12 | 9,2±0,1 | 12 | 11,5±0,3 | 12 | 10,2±0,2 | 12 | 6,8±0,1d | **09,8±0,3** |
| Symptomatic | 12 | 10,5±0,1 | 12 | 8,9±0,1 | 12 | 10,5±0,2 | 12 | 10,7±0,2 | 12 | 7,0±0,1 | **09,5±0,2** |
| **Average** | **36** | **11,3±0,2** | **35** | **8,8±0,2** | **36** | **11,4±0,2** | **36** | **10,5±0,1** | **36** | **7,1±0,1** |  |
| Pulpy juice (%) |  |  |  |  |  |  |  |  |  |  |  |
| Healthy | 12 | 55,4±0,3 | 11 | 58,0±0,7 | 12 | 53,2±0,4 | 12 | 54,8±0,4 | 12 | 56,1±0,3 | **55,5±0,3** |
| Asymptomatic | 12 | 56,1±0,3 | 12 | 55,9±0,4 | 12 | 54,1±0,2 | 12 | 55,6±0,4 | 12 | 57,4±0,4 | **55,8±0,2** |
| Symptomatic | 12 | 57,9±0,2 | 12 | 59,0±0,8 | 12 | 55,1±0,2 | 12 | 55,3±0,3 | 12 | 58,6±0,3 | **57,2±0,3** |
| **Average** | **36** | **56,5±0,2** | **35** | **57,6±0,4** | **36** | **54,1±0,2** | **36** | **55,2±0,2** | **36** | **57,4±0,3** |  |

| Supplementary Table 3. Physical characterization of ‘Femminello’ lemon fruits from healthy trees and from the asymptomatic and symptomatic sector of HLB-affected trees (diseased trees) collected in different harvesting seasons in southeastern São Paulo State. | | | | | | | | | | | | |
| --- | --- | --- | --- | --- | --- | --- | --- | --- | --- | --- | --- | --- |
|  | **n** | **2017** | **n** | **2018** | **n** | **2019** | **n** | **2020** | **n** | **2021** | **n** | **Media** |
| **Fruit shape^a^** | | | |  |  |  |  |  |  |  |  |  |
| Healthy | 20 | 1,1±0,03 | 16 | 1,2±0,03 | 16 | 1,2±0,02 | 16 | 1,1±0,01 | 16 | 1,2±0,01 | 84 | **1,2±0,01** |
| Asymptomatic | 20 | 1,2±0,02 | 16 | 1,2±0,02 | 16 | 1,2±0,02 | 16 | 1,1±0,01 | 16 | 1,2±0,01 | 84 | **1,2±0,01** |
| Symptomatic | 20 | 1,2±0,02 | 16 | 1,2±0,02 | 16 | 1,3±0,03 | 16 | 1,1±0,01 | 16 | 1,1±0,01 | 84 | **1,2±0,01** |
| **Average** | **60** | **1,2±0,01** | **48** | **1,2±0,01** | **48** | **1,2±0,01** | **48** | **1,1±0,01** | **48** | **1,2±0,01** |  |  |
| **Peel thickness (mm)** | | | | |  |  |  |  |  |  |  |  |
| Healthy | 20 | 5,0±0,15 | 16 | 4,1±0,13 | 16 | 5,7±0,26 | 16 | 6,3±0,28 | 16 | 4,9±0,18 | 84 | **5,2±0,12** |
| Asymptomatic | 20 | 4,8±0,14 | 16 | 4,4±0,13 | 16 | 5,6±0,24 | 16 | 5,8±0,19 | 16 | 4,9±0,15 | 84 | **5,1±0,12** |
| Symptomatic | 20 | 4,1±0,14 | 16 | 4,4±0,21 | 15 | 5,9±0,36 | 16 | 6,4±0,25 | 16 | 4,5±0,18 | 83 | **5,0±0,13** |
| **Average** | **60** | **4,7±0,1** | **48** | **4,3±0,09** | **47** | **5,7±0,16** | **48** | **6,2±0,15** | **48** | **4,8±0,11** |  |  |
| **Number of seeds** | | |  |  |  |  |  |  |  |  |  |  |
| Healthy | 20 | 9,1±0,99 | 16 | 7,2±0,59 | 16 | 5,1±0,42 | 16 | 6,9±0,63 | 16 | 6,4±0,41 | 84 | **7,0±0,35** |
| Asymptomatic | 20 | 7,7±0,87 | 16 | 6,6±0,85 | 16 | 6,9±0,62 | 16 | 8,4±0,52 | 16 | 5,3±0,39 | 84 | **7,0±0,34** |
| Symptomatic | 20 | 5,3±0,75 | 16 | 5,4±0,72 | 16 | 6,8±0,58 | 16 | 6,1±0,54 | 16 | 5,4±0,36 | 84 | **5,8±0,32** |
| **Average** | **60** | **7,4±0,54** | **48** | **6,4±0,43** | **48** | **6,3±0,33** | **48** | **7,1±0,37** | **48** | **5,7±0,25** |  |  |
| **Available oil (kg/ton)** | | | | |  |  |  |  |  |  |  |  |
| Healthy | 1 | 6,1 | 3 | 7,2±0,09 | 3 | 6,4±0,17 | 3 | 6,7±0,18 | 3 | 7,1±0,18 | 13 | **6,8±0,13** |
| Asymptomatic | 1 | 6,0 | 3 | 6,9±0,15 | 3 | 5,9±0,19 | 3 | 7,0±0,12 | 3 | 7,4±0,18 | 13 | **6,7±0,15** |
| Symptomatic | 1 | 5,1 | 3 | 6,9±0,04 | 3 | 6,5±0,19 | 3 | 6,0±0,24 | 3 | 7,8±0,11 | 13 | **6,7±0,21** |
| **Average** | **3** | **5,7±0,33** | **9** | **7,0±0,07** | **9** | **6,3±0,14** | **9** | **6,6±0,16** | **9** | **7,4±0,12** |  |  |
| **Fruit weight (g)** | | | |  |  |  |  |  |  |  |  |  |
| Healthy | 30 | 134,1±2,0 | 29 | 102,7±1,9 | 30 | 149,5±2,9 | 30 | 104,3±4,9 | 30 | 95,0±3,0 | 149 | **117,2±2,3** |
| Asymptomatic | 91 | 112,5±1,1 | 84 | 106,6±1,2 | 101 | 145,7±1,6 | 92 | 98,9±1,4d | 84 | 93,6±1,5d | 452 | **112,5±1,0** |
| Symptomatic | 76 | 126,2±1,1 | 78 | 113,7±1,3 | 114 | 130,4±1,9 | 118 | 98,7±1,4d | 120 | 98,9±1,8d | 506 | **112,4±1,0** |
| **Average** | **197** | **121,1±0,9** | **191** | **108,9±0,9** | **245** | **139,0±1,3** | **240** | **99,5±1,0** | **234** | **96,5±1,1** |  |  |

| Supplementary Table 4. Industrial quality of extracted ‘Femminello’ lemon juice from fruits collected from healthy trees and from the asymptomatic and symptomatic sector of HLB-affected trees (diseased trees) collected in different harvesting seasons in the south-southeast of São Paulo State. | | | | | | | | | | | | |
| --- | --- | --- | --- | --- | --- | --- | --- | --- | --- | --- | --- | --- |
| **Parameter** | **n** | **2017** | **n** | **2018** | **n** | **2019** | **n** | **2020** | **n** | **2021** | **n** | **Average** |
| **Acidity (%)** |  |  |  |  |  |  |  |  |  |  |  |  |
| Healthy | 12 | 5.5±0.05d | 12 | 6.6±0.06 | 12 | 6.0±0.06 | 12 | 6.3±0.04 | 12 | 6.8±0.05 | 60 | **6.2±0.06** |
| Asymptomatic | 12 | 5.4±0.04d | 12 | 6.5±0.06 | 12 | 6.0±0.02 | 12 | 6.2±0.04 | 12 | 6.9±0.22 | 60 | **6.2±0.08** |
| Symptomatic | 12 | 5.4±0.07d | 12 | 6.3±0.05 | 12 | 5.8±0.04 | 12 | 5.8±0.06 | 12 | 6.9±0.07 | 60 | **6.0±0.07** |
| **Average** | **36** | **5.4±0.03** | **36** | **6.5±0.04** | **36** | **5.9±0.03** | **36** | **6.1±0.04** | **36** | **6.8±0.08** |  |  |
| **°Brix** |  |  |  |  |  |  |  |  |  |  |  |  |
| Healthy | 12 | 8.8±0.04e | 12 | 11.2±0.13 | 12 | 9.6±0.03d | 12 | 10.1±0.07 | 12 | 13.0±0.08 | 60 | **10.5±0.19** |
| Asymptomatic | 12 | 8.5±0.06d | 10 | 11.0±0.12 | 12 | 9.2±0.07 | 12 | 9.6±0.12 | 12 | 12.7±0.09 | 58 | **10.2±0.2** |
| Symptomatic | 12 | 8.2±0.04d | 10 | 10.1±0.15 | 12 | 9.1±0.06 | 12 | 8.8±0.08 | 12 | 13.3±0.27 | 58 | **9.9±0.25** |
| **Average** | **36** | **8.5±0.05** | **32** | **10.8±0.12** | **36** | **9.3±0.05** | **36** | **9.5±0.10** | **36** | **13.0±0.10** |  |  |
| **Ratio** |  |  |  |  |  |  |  |  |  |  |  |  |
| Healthy | 12 | 1.6±0.01 | 12 | 1.7±0.02 | 12 | 1.6±0.02 | 12 | 1.6±0.01 | 12 | 1.9±0.01 | 60 | **1.7±0.02** |
| Asymptomatic | 12 | 1.6±0.01 | 12 | 1.7±0.02 | 12 | 1.5±0.01 | 12 | 1.5±0.02 | 12 | 1.9±0.04 | 60 | **1.6±0.02** |
| Symptomatic | 12 | 1.5±0.02 | 11 | 1.6±0.02 | 12 | 1.6±0.02 | 12 | 1.5±0.01 | 12 | 1.9±0.04 | 59 | **1.6±0.02** |
| **Average** | **36** | **1.6±0.01** | **35** | **1.7±0.01** | **36** | **1.6±0.01** | **36** | **1.6±0.01** | **36** | **1.9±0.02** |  |  |
| **Oils (%)** |  |  |  |  |  |  |  |  |  |  |  |  |
| Healthy | 4 | 0.03±0.00d | 4 | 0.07±0.00 | 5 | 0.05±0.00 | 4 | 0.07±0.01 | 4 | 0.11±0.01 | 21 | **0.07±0.01** |
| Asymptomatic | 4 | 0.04±0.00d | 4 | 0.06±0.00 | 5 | 0.05±0.00d | 4 | 0.08±0.00 | 4 | 0.13±0.01 | 21 | **0.07±0.01** |
| Symptomatic | 4 | 0.05±0.00d | 4 | 0.06±0.00d | 4 | 0.08±0.00 | 4 | 0.07±0.00 | 4 | 0.15±0.01 | 20 | **0.08±0.01** |
| **Average** | **12** | **0.04±0.00** | **12** | **0.06±0.00** | **14** | **0.06±0.00** | **12** | **0.08±0.00** | **12** | **0.13±0.01** |  |  |
| **Viscosity (cP)** |  |  |  |  |  |  |  |  |  |  |  |  |
| Healthy | 11 | 6.3±0.22 | 4 | 6.4±0.17 | 12 | 5.2±0.24 | 12 | 4.9±0.23 | 12 | 8.6±0.17 | 51 | **6.3±0.22** |
| Asymptomatic | 10 | 6.1±0.15 | 4 | 5.1±0.08 | 12 | 3.6±0.08d | 12 | 6.7±0.25 | 12 | 9.2±0.10 | 50 | **6.3±0.29** |
| Symptomatic | 11 | 6.1±0.26 | 4 | 4.3±0.02d | 12 | 5.1±0.08 | 12 | 4.7±0.05d | 12 | 8.0±0.10 | 51 | **5.8±0.20** |
| **Average** | **32** | **6.2±0.12** | **12** | **5.2±0.27** | **36** | **4.6±0.15** | **36** | **5.4±0.19** | **36** | **8.6±0.11** |  |  |
| **Hesperidin (mg/kg)** |  |  |  |  |  |  |  |  |  |  |  |  |
| Healthy | 12 | 461.6±17.02 | 4 | 355.1±26.75 | 12 | 93.6±1.71d | 12 | 292.4±5.42 | 12 | 392±3.79d | 52 | **313.4±19.25** |
| Asymptomatic | 12 | 471.3±18.79 | 4 | 519.5±38.16 | 12 | 106.4±3.77d | 12 | 394.9±13.80 | 12 | 330.2±5.61 | 52 | **340.6±20.58** |
| Symptomatic | 12 | 514.8±13.87 | 4 | 379.4±49.28 | 12 | 151.0±9.74 | 12 | 399.5±18.39 | 12 | 418.7±11.71 | 52 | **371.6±19.38** |
| **Average** | **36** | **482.6±10.14** | **12** | **418±29.92** | **36** | **117±5.39** | **36** | **362.3±11.32** | **36** | **380.3±7.65** |  |  |
| **Pectin (mg/kg)** |  |  |  |  |  |  |  |  |  |  |  |  |
| Healthy | 5 | 405.2±16.69 | 4 | 220.0±24.09 | 5 | 306.1±27.89 | 4 | 317.9±20.74 | 4 | 302.1±26.34 | 22 | **314.4±16.08** |
| Asymptomatic | 5 | 435.0±14.20 | 4 | 193.0±8.26 | 5 | 308.8±21.29 | 4 | 329.6±32.69 | 4 | 273.1±9.39 | 22 | **313.7±19.04** |
| Symptomatic | 5 | 403.9±29.12 | 4 | 217.6±18.76 | 5 | 363.9±20.32 | 4 | 362.1±34.14 | 4 | 208.4±43.48 | 22 | **317.8±21.30** |
| **Average** | **15** | **414.7±11.89** | **12** | **210.2±10.23** | **15** | **326.3±14.39** | **12** | **336.6±16.55** | **12** | **261.2±19.56** |  |  |
| **Limonin (mg/kg)** |  |  |  |  |  |  |  |  |  |  |  |  |
| Healthy | 5 | 25.5±1.08 | 4 | 21.3±3.66 | 5 | 23.3±0.66 | 4 | 16.9±1.35 | 4 | 9.8±0.05 | 22 | **19.8±1.38** |
| Asymptomatic | 5 | 31.9±1.29 | 4 | 30.6±1.59 | 5 | 29.0±2.41 | 4 | 19.0±1.72 | 4 | 10.9±0.61 | 22 | **24.8±1.86** |
| Symptomatic | 5 | 29.9±1.63 | 4 | 32.4±5.82 | 5 | 27.5±3.82 | 4 | 32.9±5.65 | 4 | 13.0±0.89 | 22 | **27.2±2.19** |
| **Average** | **15** | **29.1±1.02** | **12** | **28.1±2.58** | **15** | **26.6±1.55** | **12** | **22.9±2.81** | **12** | **11.2±0.51** |  |  |
